# Computational Screening of First-Row Transition-Metal Based Alloy Catalysts—Ligand Induced N_2_ Reduction Reaction Selectivity

**DOI:** 10.1021/acsphyschemau.1c00021

**Published:** 2021-11-29

**Authors:** Arunendu Das, Shyama Charan Mandal, Akhil S. Nair, Biswarup Pathak

**Affiliations:** † Department of Chemistry, Indian Institute of Technology Indore, Indore, 453552, India

**Keywords:** nitrogen reduction reaction, alloy-based catalysts, density functional theory, potential determining step, overpotential

## Abstract

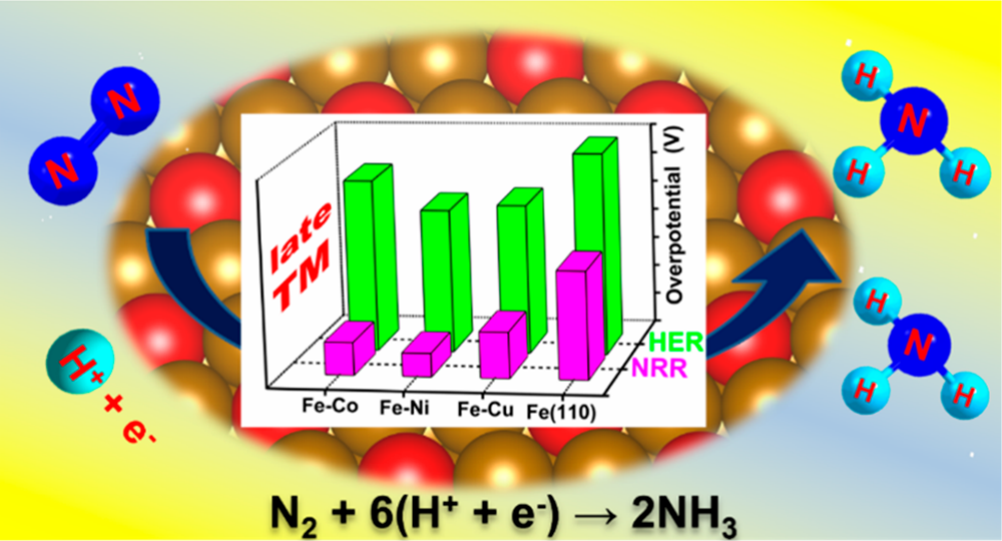

Large-scale ammonia production through sustainable strategies from naturally abundant N_2_ under ambient conditions represents a major challenge from a future perspective. Ammonia is one of the promising carbon-free alternative energy carriers. The high energy required for N≡N bond dissociation during the Haber-Bosch process demands extreme reaction conditions. This problem could be circumvented by tuning Fe catalyst composition with the help of an induced ligand effect on the surface. In this work, we utilized density functional theory calculations on the Fe(110) surface alloyed with first-row transition-metal (TM) series (Fe–TM) to understand the catalytic activity that facilitates the electrochemical nitrogen reduction reaction (NRR). We also calculated the selectivity against the competitive hydrogen evolution reaction (HER) under electrochemical conditions. The calculated results are compared with those from earlier reports on the periodic Fe(110) and Fe(111) surfaces, and also on the (110) surface of the Fe_85_ nanocluster. Surface alloying with late TMs (Co, Ni, Cu) shows an improved NRR activity, whereas the low exchange current density observed for Fe–Co indicates less HER activity among them. Considering various governing factors, Fe-based alloys with Co (Fe–Co) showed enhanced overall performance compared to the periodic surface as well as other pure iron-based structures previously reported. Therefore, the iron-alloy based structured catalysts may also provide more opportunities in the future for enhancing NRR performance via electrochemical reduction pathways.

## 1 Introduction

N_2_ fixation converts the most abundant molecule in the earth’s atmosphere, dinitrogen (N_2_), to ammonia (NH_3_), which has received attention as a carbon-free alternative energy carrier.^1−4^ Moreover, ammonia production plays an indispensable role in determining the strength of an economy as it is used to produce fertilizers and other synthetic chemicals such as explosives, dyes, and resin, among others.^5,6^ Currently, 500 million tons of NH_3_ are being produced per year from gaseous N_2_ and H_2_ via the industrial Haber-Bosch (HB) process through *N_2_ dissociation.^7,8^ Nevertheless, ammonia production demands harsh reaction conditions with high temperature (∼700 K) and pressure (∼100 bar) even after using promoters on active Fe/Ru metal-based catalysts.^9^ However, the biological N_2_ fixation by nitrogenase enzyme via the proton-coupled electron transfer (PCET) process, that is, electrochemical N_2_ reduction reaction (NRR, N_2_ + 6H^+^ + 6e^–^ → 2NH_3_), has recently emerged as a highly desirable alternative process that can be performed under ambient conditions.^10^ The electrochemical NRR can be initiated through associative adsorption via N_2_ hydrogenation (an associative mechanism) to form *NNH instead of direct N≡N dissociation.^11^ Large scale ammonia synthesis through electrochemical pathways may be an issue and would need to be developed for the industrialization of the process; hence, ammonia synthesis by an artificial electrochemical system could be a promising process in the future with plentiful renewable energy sources, avoiding the extreme conditions now being used. Several experimental and theoretical studies have shown that molecular and heterogeneous catalysts containing Fe, Co, and Mo metals are widely used for better activation of the inert N_2_ with an improved NRR efficiency.^12−17^ However, other possibilities have been discussed in previous theoretical studies for ammonia synthesis under ambient conditions, showing higher favorability toward *NNH formation instead of direct dissociation upon *N_2_ adsorption.^18−27^ It was also reported earlier that the *NNH formation following an associative mechanism is much faster on the active site of the Fe_3_/θ-Al_2_O_3_(010) system and therefore bypasses the balance between direct N≡N dissociation and *NH_*x*_ desorption with increased NRR activity compared to the pure Fe surfaces.^18^ In most of the cases, the formation of *NNH on the considered Fe-based catalyst surface are endergonic in nature.^24−27^ Therefore, inducing the active site of the catalyst by alloying can be an effective strategy that improves *NNH formation, achieving the ammonia synthesis at ambient pressure and temperature. Moreover, the yield of ammonia produced from NRR can be improved by regulating the potential, pH, electrolyte, etc., that operate under an electrochemical condition.^28^ Nishibayashi and co-workers reported Fe, Co, and Mo based molecular electrocatalysts with an anionic PNP-pincer ligand showing enhanced catalytic activity toward NRR and also ensuring the catalyst stability under mild reaction conditions.^13,14^ But long-lasting heterogeneous electrocatalysts can be more easily incorporated into the fuel cell compared to the aforementioned molecular catalysts.^15,16^ Besides, the activity on a heterogeneous catalyst can be tuned by inducing ligand effects to change its surface electronic properties.^29−32^ The ligand effects are beneficial for constructing a tunable d-orbital electron which alleviates the slow kinetics for *N_2_ activation followed by hydrogenation. It also tunes the well-defined active sites on the catalytic surface, which are principal in regulating the NRR activity. However, the competitive hydrogen evaluation reaction (HER) instead of ammonia synthesis is another reaction occurring under the same reaction conditions, and hence, a low Faradaic efficiency of NH_3_ is observed (FE ∼ <1%).^28,33,34^ Several theoretical studies have predicted that the catalytic activity for most pure metals and their oxide/nitride-based catalysts surface with low index sites prefers HER rather than NRR, and hence these are associated with low ammonia production efficiency.^20−22,35,36^ Besides, ammonia production efficiency can also be low with an easy H^+^ reduction, as the induction of applied potential increases the HER rate that operates during an electrochemical reaction. Varieties of electrode materials such as Pt, Ru, Cu, Fe, and Ni have been demonstrated for electrochemical ammonia production. It has been suggested that Fe, Co, and Ni materials could be helpful in improving the catalyst stability and high activity toward NRR instead of HER.^37−42^

In the literature, bimetallic catalysts are exposed to extensive scrutiny with respect to heterogeneous surface catalysis due to the flexible choice of composition.^28,43^ Therefore, the development of an alloy-based heterogeneous electrocatalyst might display NRR activity with several degrees of enhancement as compared to pure iron based electrocatalysts.^26,27^ The NRR activity can be tuned by regulating the binding strength of some of the important NRR intermediate species on the surface-active center of the catalyst compared to the pure surface. Especially, the weaker binding species can be removed easily from the surface-active site and prevent the blocking of active sites that are available for N_2_ adsorption. Henceforth, the longevity of an alloy-based iron electrocatalyst can be enhanced by lessening the catalytic surface that is poisoned. So far, Fe, Ni, and Fe–Ni nanoparticles over Pt black have been synthesized with improved efficiency for NRR through an alkaline exchange membrane (AEM)-based electrolyzer cell in research conducted by National Institute of Standards and Technology (NIST) and Colorado School of Mines (CSM) team.^42^ The primary focus in previous studies has been on the challenges of achieving both high activity and selectivity in the development of iron-based catalyst material.^18,25−27,42^

Inspired by all these, we performed a first-principle-based computational screening study of NRR activity of iron-based alloy catalysts to understand the origin of improvement in catalytic reactivity as well as selectivity for NRR. For the screening, we have considered Fe(110) surface alloyed Fe–TM with first-row transition series metals (TM = Sc, Ti, V, Cr, Mn, Co, Ni, and Cu) and scrutinized the NRR activity for all the designed alloys along with a detailed activity comparison between the Fe–TM and pure Fe(110) surface catalysts. Specifically, we attempt to understand the binding strength of the *N_2_H_*x*_ and *NH_*x*_ intermediate species on the Fe–TM alloy-based surfaces as these are the decisive intermediates in NRR. Moreover, both dissociative and associative mechanistic pathways for NRR are investigated, and the energy changes associated with all the elementary steps of NRR are analyzed. We have also addressed the overpotential associated with HER and NRR, determining the catalyst activity and selectivity. Alloys constructed with late 3d metals (Fe–TM; TM = Co, Ni, and Cu) result in significant activity differences due to various factors which are discerned in the study.

## 2 Computational Details

**Scheme 1:**
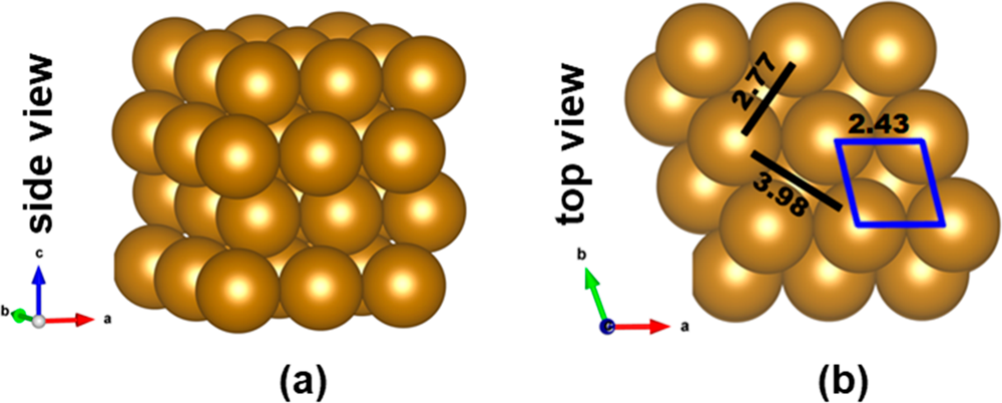
(a) Side and (b) Top Views of Optimized Fe(110) Surface (Four-Layers) with Fe–Fe Bond Lengths Values in Å

All the density functional theory (DFT) calculations are carried out with the projector augmented wave (PAW) method using the Vienna ab initio simulation package (VASP).^44−46^ The electron exchange-correlation functional is described through generalized gradient approximation (GGA) with the Perdew–Burke–Ernzerhof (PBE) functional.^47^ A plane-wave basis set with a kinetic energy cutoff of 500 eV is adopted to expand the electronic wave functions. The periodic Fe(110) surface composed of four atomic layers is modeled with a 3 × 3 supercell consisting of 36 atoms by using a slab model, as shown in Scheme 1. During optimization, the two top layers are relaxed, and the bottom two layers are fixed. The Brillouin zone is sampled with a set of (3 × 3 × 1) Monkhorst–Pack k-point grids for periodic calculations. A 15 Å vacuum along the *Z* direction is employed to avoid the periodic interactions between adjacent layers. The convergence criteria of all the relaxed atomic coordinates were 10^–4^ eV and 0.02 eV/Å for total energy and the Hellman–Feynman force, respectively during optimization of the catalysts and intermediates. The spin-polarized calculations are performed for all the considered geometries. The van der Waals interactions were treated using Grimme’s DFT-D3 method.^48^ The calculated values of magnetic moment, lattice parameter for bulk Fe bcc structure, and work-function (ϕ) for our modeled Fe(110) are in very much closer agreement with earlier reports.^49−52^ The Hubbard potential calculation has been avoided in this work as practiced in other Fe-based catalyst surface studies as there are negligible changes in reaction free energy (Δ*G*) values observed in the potential determining step (PDS) between DFT-GGA and DFT-U calculation for the periodic Fe(111), as reported previously from our group.^18,20−27,52^ A 9 × 9 × 1 k-point grid is used for alloy-based surfaces considered in our study to examine electronic structure. The zero-point energy (ZPE) correction is calculated from the observed vibrational frequencies (*v*_*i*_) which were obtained from the density functional perturbation theory (DFPT) method as shown in eq 1,
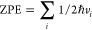
1where *v*_*i*_ and ℏ are the frequency of the *i*th vibrational mode and Planck constant, respectively. The adsorption energies (*E*_ad_) are calculated for all possible NRR intermediate species using the following equation:
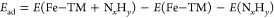
2where *E*(Fe–TM + N_*x*_H_*y*_) is the total energy of the iron-alloy (TM = Sc to Cu) based catalyst with adsorbate, *E*(Fe–TM) and *E*(N_*x*_H_*y*_) are the total energies of the periodic system alloying with first-row TM series over the surface and *N_*x*_H_*y*_ intermediate species in the optimized geometry. The adsorbed intermediate species over the most favorable active site of their surface have been denoted with an asterisk (∗) sign. All the reaction free energy changes (Δ*G*) of the consecutive steps are calculated by using the computational hydrogen electrode (CHE) model proposed by Nørskov et al., and the equation can be represented as
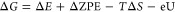
3where Δ*E*, ΔZPE, Δ*S*, are the changes in total energy, zero-point energy, and entropy, and eU is the applied potential for an elementary electrochemical reaction step, respectively.^53,54^ For gaseous species, their entropy value has been taken from ref (55), and the entropy correction for an adsorbed species is not included here.^55^ Moreover, Bader atomic charges were calculated for some of the important intermediates adsorbed over Fe–TM using the Henkelman code with the near-grid algorithm refine-edge method.^56−58^

## 3 Result and Discussion

### 3.1 Material Modeling

The optimized iron bulk bcc structure is found to have a lattice constant and magnetic moment of 2.81 Å and 2.16 μB, respectively. Initially, the Fe(110) surface was modeled from a bulk bcc structure, given in Scheme 1.

**Figure 1:**
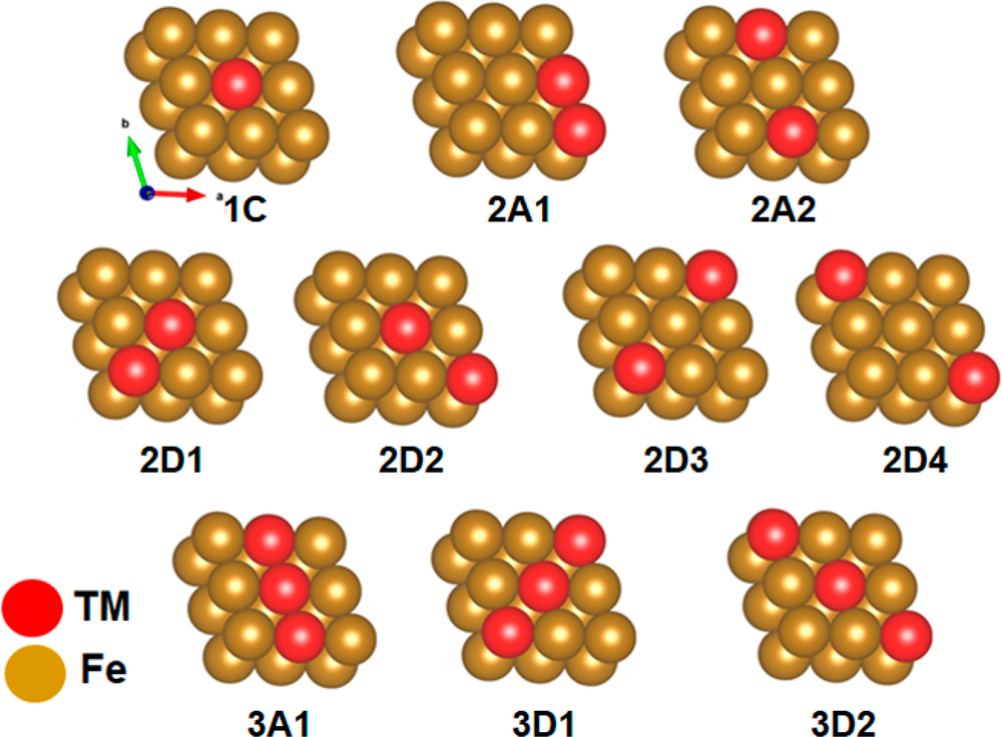
Construction of Fe-based surface alloys (Fe–TM) from a periodic Fe(110) surface (host) by replacing top layer atoms by guest TM metals. The digits 1, 2, and 3 in the labels represent the number of TM atoms substituting Fe and the letters A, C, and D represent adjacent, center and diagonal wise substitutions, respectively.

Now, the bimetallic Fe–TM alloy surfaces are constructed by replacing the one, two, and three Fe atoms of the periodic Fe(110) surface with the corresponding first-row TM atoms to build the model (Figure 1) with different possibilities.

Our calculated total energies show that modeled 1C, 2A1, 2D2, 3A1, and 3D2 are lower/similar in energy compared with their respective possibilities in the majority of alloys with TM (Table S1). Hence, 1C, 2A1, 2D2, 3A1, and 3D2 have been used for further study. Next, we calculated the work-function and surface energy for each of the cases. The details of the work-function and surface energy calculations can be found in Text-S1 of the Supporting Information. Figure S1a shows that the work-function for TM-substituted alloys are lower or nearer those of the calculated work-function for the pure Fe(110) surface. In most of the cases, the work function values decreased with increasing TM content except for V and Cr-based surface alloys. In the case of the Fe–Cr surface alloy, a significant jump in the work-function values was observed with an increase in the content of diagonal Cr substitution (model 3D2) on the Fe(110) surface. For late-TM (Co, Ni, and Cu) based alloys, the work function value changes negligibly when the TM content increases. Besides, a slight decrease has been observed in the surface energy (Figure S1b) with increasing TM content for Co and Ni-based surface alloys. However, substitution with the Mn and Cu (model 3D2) results in an increase in surface energy showing the largest value on Fe–Mn and Fe–Cu among all the Fe–TM surface alloys. A steady decrease in the surface energies with increasing early-TM (Sc to Cr) content was observed for each of the Fe-based surface alloys. In addition, it is interesting to note that Fe-based surface alloys considering model 3D2 for both Fe–Co and Fe–Ni showed similar surface energies. Generally, metal surfaces with high surface energy and low work function values are considered highly reactive surfaces and can be unstable under electrochemical conditions. Therefore, one needs to be very careful regarding the choice of catalyst that solely depends on the surface energy. Since the bulk structures of many first-row transition metals are nonbcc, we have considered the respective bulk unit cell of these elements for the calculation of average binding energy and formation energy to screen the Fe–TM alloys based on their stability.^59,60^ The formation energy (*E*_f_) and average binding energy (*E*_b_) of the considered systems are calculated for their energetic stability as follows:
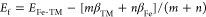
4
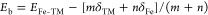
5where *E*_Fe-TM_ is the optimized energy of the surface alloyed with TM, *m* and *n* are the number of iron and TM atoms present in the systems, δ stands for the energy of an isolated atom, and β stands for the bulk energy per atom with their respective crystal structure. The less is the formation energy, the higher would be the thermodynamic stability of the system. The average binding energy per atom (*E*_b_) for the Fe-rich iron-alloy (Fe–TM) for all considered models (1C, 2A1, 2D2, 3A1, and 3D2) is shown in Figure S2 determined using eq 5. A more negative binding energy indicates a higher stability of iron-alloy based surface structure catalysts in comparison with the isolated states of their atoms. Therefore, all the Fe–TM alloys incorporate TM atoms diagonally (3D2 model) over Fe(110) except for Cr-, Mn-, and Cu-based surface alloys which have been found to bind strongly compared to the periodic Fe(110) surface. In addition, the calculated formation energy for each of the 3D2 models also supports our finding (Figure S3a), possessing the required thermodynamic stability to be useful as catalyst materials. Henceforth, the model 3D2 for Fe–TM alloy systems among the entire set of possible models is considered for further catalytic studies.

### 3.2 Adsorption of Different Intermediates

**Figure 2:**
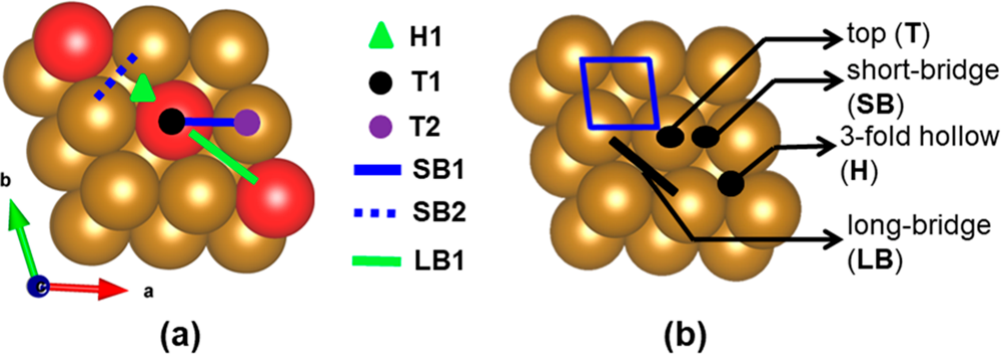
Adsorption sites for (a) Fe–TM alloys and (b) pure Fe(110) surface, respectively. The adsorption sites of the pure Fe(110) surface are presented from earlier reports.^61,62^

The important intermediates *N, *NH, *NH_2_, *NH_3_, *NNH, *NNH_2_ involved in the NRR mechanism are scrutinized for their adsorption on the Fe–TM alloys and pure Fe(110) surface. All the possible adsorption sites are shown in Figure 2, and the most stable sites have been considered for the energetic study. The adsorption energy of each of the intermediate species for the Fe–TM alloys (model 3D2) and pure Fe(110) surface are given in Table 1.

**Table 1:** Adsorption Energies (*E*_ad_) with Preferred Adsorption Sites (in Parentheses) for All Possible NRR Intermediates on the Fe–TM Surface Alloys Structure and Periodic Fe(110) Surface^26^

	*E*_ad_ (adsorption energy in eV with preferred adsorption site in the parentheses)							
systems	*N (H1)	*NH (H1)	*NH_2_(SB2)	*NH_3_(T1)	*N_2_(LB1)	*NNH (LB1)	*NNH_2_(LB1)	*H (H1)
Fe–Sc	–7.20	–6.02	–3.48	–1.11	–1.22	–4.86	–4.66	–3.03
Fe–Ti	–6.97	–6.12	–3.56	–1.11	–1.32	–4.97	–4.72	–3.14
Fe–V	–6.81	–6.13	–3.66	–1.28	–1.33	–4.90	–4.47	–3.07
Fe–Cr	–6.67	–5.89	–3.60	–1.19	–1.13	–4.67	–4.13	–2.97
Fe–Mn	–7.09	–5.96	–3.41	–1.20	–1.52	–4.81	–4.29	–3.08
Fe–Co	–6.09	–5.24	–3.23	–0.87	–0.70	–3.95	–3.54	–2.96
Fe–Ni	–5.93	–5.10	–3.13	–0.83	–0.39	–3.72	–3.40	–2.87
Fe–Cu	–5.90	–5.06	–3.28	–0.69	–0.05	–3.45	–3.30	–2.88
Fe(110)^26^	–6.57 (H)	–5.57 (H)	–3.37 (H)	–1.00 (T)	–1.07 (LB)	–4.32 (LB)	–3.78 (H)	–3.06 (H)

**Figure 3:**
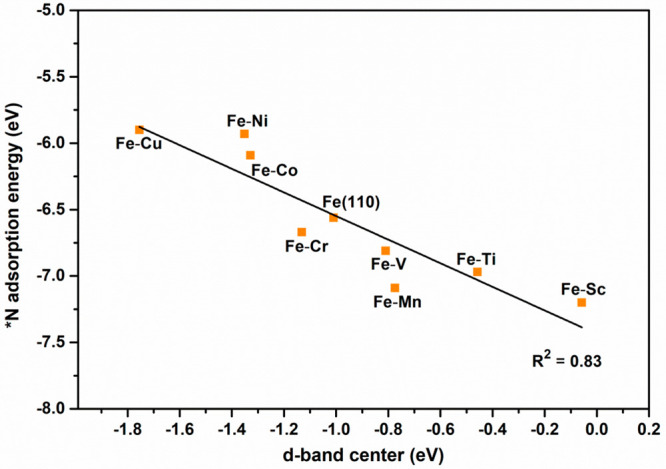
*N adsorption energy plotted against the d-band center position of the surface constituting Fe and TM atoms of the catalysts.

Furthermore, we have compared our results with the previously reported results of the periodic Fe(110) surface, given in Table 1. The adsorption sites over alloys for most of the NRR intermediate species are found to be same as that of pure surface. Interestingly, we have observed that the surface alloying with early TM (Sc to Mn) exhibits high adsorption energy for *N mediated species in comparison to those of the periodic Fe(110) surface. However, the Fe–TM alloying with late TM (Co to Cu) exhibits a wide range of adsorption energies with an overall tendency to decrease its binding with respect to the periodic Fe(110) surface. The adsorption behavior of each of the considered NRR intermediate species for Fe–Co and Fe–Ni alloys is given in Table S2 with structural parameters of the optimized catalyst surface (Table S3). Therefore, the Fe–TM surface with early TM placed in the strong binding regime, and late-TM alloys placed in the relatively weaker binding regime. To understand the reason behind the observed binding strength trends of adsorbed *N over Fe–TM, we examined the d-band center position of the catalyst surface (Figure 3). The calculated d-band center values for late-TM based alloys downshifted toward the negative direction, allowing *N to bind weakly (Table 1) compared to pure Fe(110) and early-TM (Fe–TM) surface alloys as well.

It is also obvious from the values obtained from Table 1 and Figure S4 that the general scaling between NRR intermediates is highly followed for Fe–TM alloys with high consistency (*R*^2^ = 91) in *NNH/*NH. However, low coefficient determination (*R*^2^ = 51) values indicate poor scaling or wider disparity in *NH_2_ with reference to *N binding. For example, the Fe–Cu alloy is showing the weakest *N adsorption energy owing to the minimum adsorption strength toward *NNH/*NH in the series. From Table S4, we have also observed a wider range of adsorption energy values for coadsorption of two *N (dissociative adsorption) while substituting with early and late TM in different compositions. It indicates that the strength of interaction between surface atoms (Fe, TM) and adsorbed species is sensitive to chemical composition and catalyst’s structural parameters of the Fe–TM, given in Tables S2–S4. Hence, reaction free energies (Δ*G*) associated with the consecutive reaction steps following the most stable adsorbate geometries are investigated. The details will be discussed in the following section to understand different NRR activities of these Fe–TM alloys.

### 3.3 NRR Mechanism

**Scheme 2:**
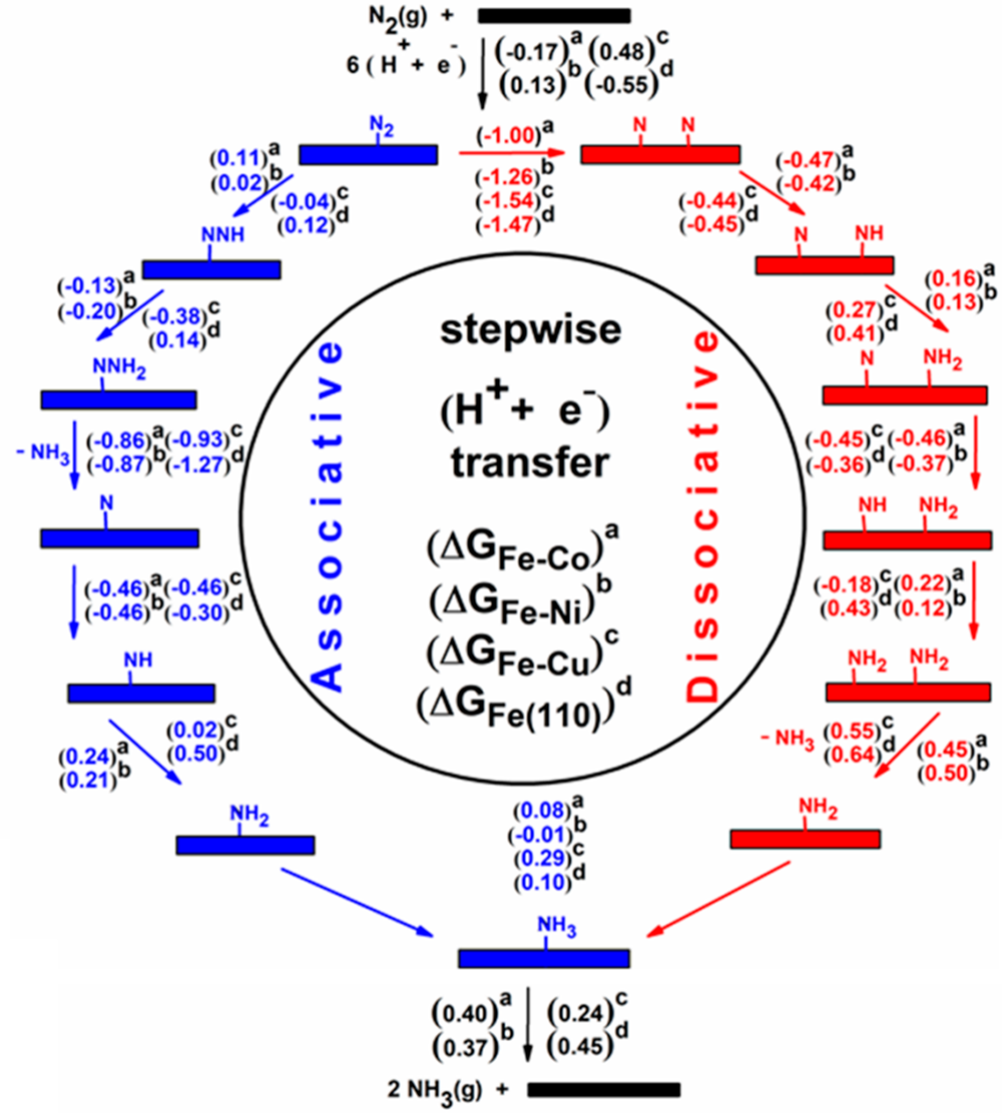
Reaction Steps Involved in the Associative and Dissociative NRR with Reaction Free Energy Change Values (Δ*G* in eV) on Fe–TM (TM = Co, Ni, and Cu) Alloys and Periodic Fe(110) Surface Δ*G* values are given in the parentheses for Fe–Co, Fe–Ni, Fe–Cu, and periodic Fe (110) which are labelled as a, b, c, and d, respectively.

The free energy diagrams have been widely used to explain reaction free energy changes (Δ*G*) associated with adsorbed intermediates involved in each of the elementary steps for any reaction. Along with the free energy analysis, we have considered the effect of applied potential on the elementary reactions associated with NRR, occurring under the electrical potential in the real scenario. NRR has been proposed to occur through two different mechanisms: (a) dissociative, where the N≡N bond directly dissociates to form 2*N, (b) associative, where *NNH is formed through post-*N_2_-adsorption by attacking first H^+^, and therefore, successive protonation in each pathway leads to the formation of ammonia as a final product. The elementary steps with the largest positive Δ*G*_max_ value are identified in each following pathway, considered as potential determining steps. The NRR occurs through the formation of *NNH_2_, as a result of *NNH protonation via a distal associative mechanistic pathway. The *NHNH formation in an alternating associative mechanism has been avoided in our current study as it was found to be a less favorable pathway over the periodic Fe(110) surface, as reported in our earlier studies.^26^ However, the free energy profile for both associative and dissociative mechanisms have been adapted in this study along with their Δ*G* values (Scheme 2, Figure S5 and Figure S6). The Δ*G*_max_ values for dissociative and associative pathways, working potential (*U*_work_), and overpotential (η_NRR_) for Fe–TM alloys are given in Table S5 and Table S6 and also compared to previously reported Fe(110) surface values as well.^26^

**Figure 4:**
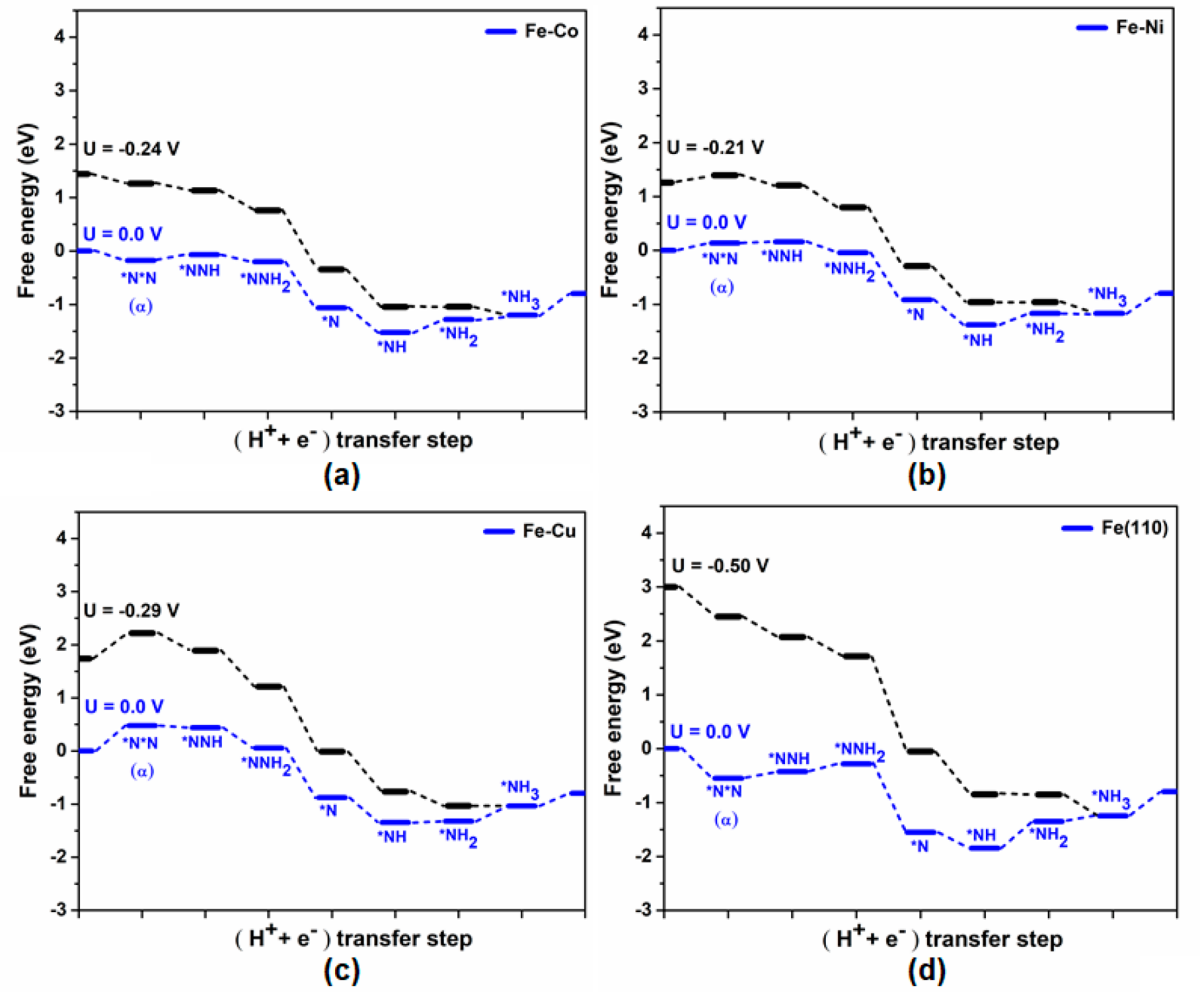
Free energy analysis for NRR following associative mechanistic pathways for (a) Fe–Co, (b) Fe–Ni, (c) Fe–Cu alloys, and (d) periodic Fe(110) surface, respectively.^26^

It can be seen that the favorability of direct *N_2_ dissociation forming the 2*N, following the dissociative mechanistic pathway, decreases while moving from the periodic Fe(110) surface alloying with early to late TM. However, *NNH formation with Δ*G*_max_ associated with PDS is improved significantly following the associative mechanistic pathway for the same. Furthermore, the free energy diagram (Figure S5) predicts that *NH_2__*NH_2_ + (H^+^ + e^–^) → *NH_2_ + NH_3_ (g) step is the PDS with the largest positive Δ*G*_max_ values (Table S5) for NRR occurring on an individual surface alloy, except for Fe–TM (TM = Sc, V, Cr) where *NH_*NH_2_ + (H^+^ + e^–^) → *NH_2__*NH_2_ is the PDS for the dissociative pathway. In the case of the associative pathway, we found different reaction steps with Δ*G*_max_ for the surface alloying with Cu (Fe–Cu), among others (Figure S6 and Table S6). Therefore, we have observed that the calculated working potential (*U*_work_) values following the dissociative pathway are higher than that of the associative pathway (Figure S7). In addition, the observed PDS and working potential values linked with the associative pathway for surface alloying with late TM are lower compared to those with early TM and the periodic Fe(110) surface. Consequently, the lower negative working potential values turn out to be less of an overpotential for the late-TM based Fe alloy surfaces implying an enhanced activity toward NRR. Furthermore, we have checked the binding energy of *N mediated species as the observed weaker binding of *N mediated species on late-TM based Fe alloys can lessen their catalyst surface from poisoning compared to the Fe(110) surface alloy and early-TM based Fe alloy (Table 1). Figure 4 shows the effect of applied potential on the free energy changes associated with an elementary step following a favorable associative mechanistic pathway for Fe(110) and late-TM surface alloys. To illustrate overpotential calculations, we have followed the CHE model proposed by Nørskov and co-workers.^53,54^ The corresponding working potential (*U*_work_) observed for the Fe–Co, Fe–Ni, and Fe–Cu are −0.24 V, −0.21 V, and −0.29 V, respectively, which are less in comparison to that for the pure Fe(110) surface (−0.50 V), given in Figure 4. Moreover, the calculated overpotential value for Fe–Ni (0.08 V) is found to be least compared to those for Fe–Co (0.11 V) and Fe–Cu (0.16 V). Therefore, Fe–Ni shows a relatively higher activity toward NRR in comparison to the periodic Fe(110) surface (overpotential = 0.37 V) which also supports the previous studies reported by Renner and co-workers.^42^ However, we did not consider pH effects in our current study. From the literature study, we understood that the calculated value of the overpotential remains almost the same with changing the pH of the reaction environment.^23^

### 3.4 HER Activity

**Figure 5:**
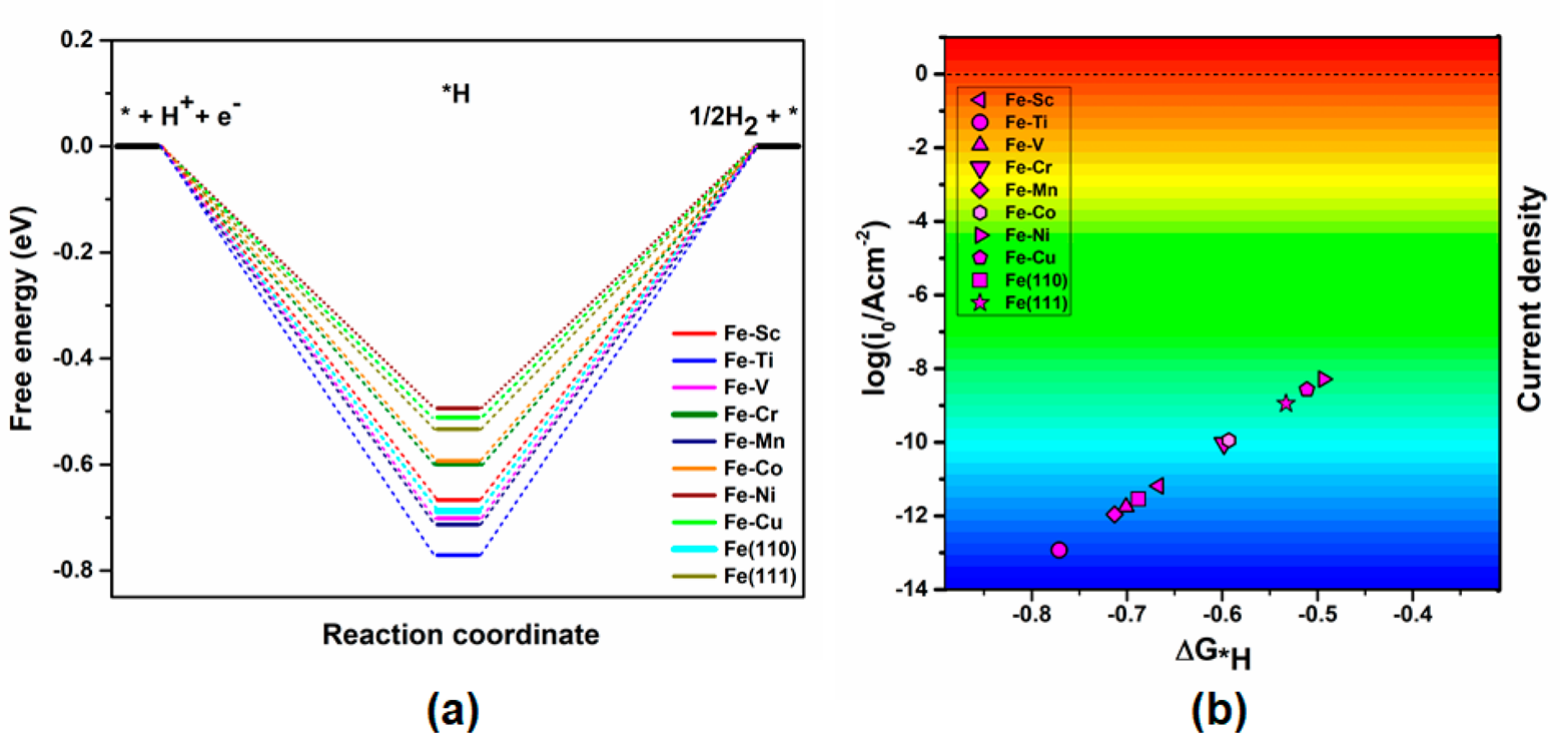
(a) Free energy analysis of 2e^–^ reduction for HER on Fe–TM alloys, pure surface, and the earlier studied NC-based catalyst. (b) Current density and HER activity relationship of considered alloys and periodic surfaces represented with color gradient by correlating G_*H_ and current density (*i*_0_) expressed in logarithmic values. HER activity is marked in ascending order while moving from the blue to the red region.^26,27,63,64^

A competitive pathway for the 6e^–^ NRR with NH_3_ formation is the 2e^–^ reduction of protons resulting in H_2_ formation. Apart from the NRR, we have investigated this hydrogen evolution reaction (HER) activity of Fe–TM alloys by free energy analysis. In the Heyrovsky-type mechanistic pathway (Text S2), the proton from the solution is adsorbed into the catalytic active site for *H formation and later desorbed, forming H_2_. Therefore, we are considering HER involving two elementary (R1, R2) steps of the Heyrovsky-type mechanism. Figure 5a represents the HER free energy diagram of 2e^–^ reduction. From the diagram, we have understood that the combination of adsorbed *H with protons to form H_2_ was observed as the rate-determining step (RDS) for Fe–TM alloys. There is variation in the activity determining reaction for the *H formation and protonation itself in the next step along with the Fe–TM series. Moreover, the current densities of each system for the HER process have been calculated using the following equation as suggested by Nørskov et al.,
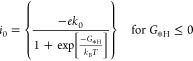
6where *k*_0_ is the rate constant, which has been set to be 1 in earlier studies for HER activity, *k*_B_ is the Boltzmann constant, G_*H_ is the reaction free energy of the *H adsorption and *T* is the temperature.^63,64^ Since ΔG_*H_ is the only activity descriptor involved in the HER process, the exchange current density vs Gibbs free energy of adsorption is also plotted in Figure 5b. Among all of them, the NRR active Fe–Ni and Fe–Cu alloys (Table S7) show prominent activity toward HER, whereas Fe–Co appears to be a less HER active candidate. Our calculated current density plot also shows that Fe–Co can be a promising catalyst for NRR owing to the low exchange current density associated with HER compared to the Fe–Ni and Fe–Cu alloys. It occurs due to the weak and strong adsorption of the *H (Table 1) to the active site of the surface alloys.

### 3.5 NRR vs HER

**Figure 6:**
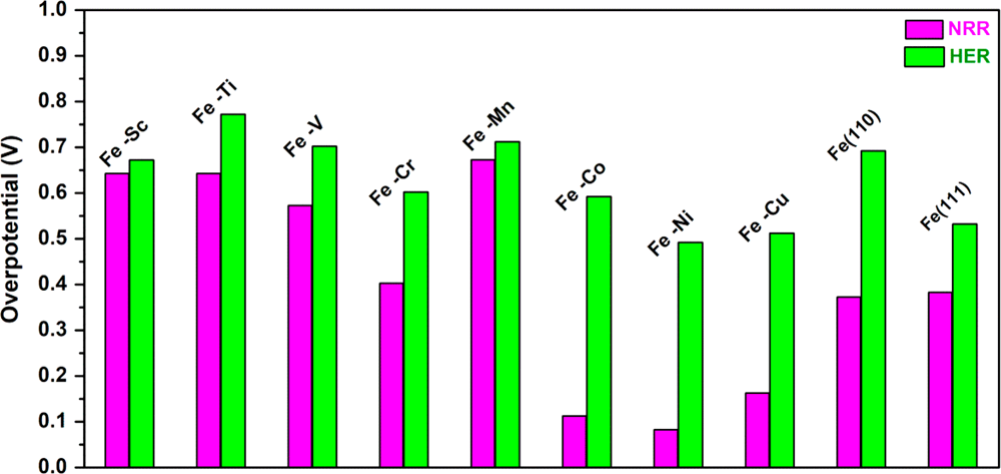
Comparative study of the overpotential for NRR and HER on considered sites of the Fe–TM alloys and other Fe-based catalysts studied previously.^26^

Both NRR and HER overpotential together are plotted in Figure 6. From this figure, we can observe that all the Fe–TM surface alloy catalysts exhibit high selectivity toward NH_3_ formation rather than hydrogen evolution. We notice that the overpotential associated with HER on the surface alloyed with late TM shows large disparity from NRR overpotential compared to pure Fe(110), Fe(111) surfaces, and early-TM based alloys. Therefore, NH_3_ product selectivity reaches the maximum in comparison to that of alloying with early TM and other considered systems. So, the Fe–TM alloying with late TM can be considered as promising catalysts that offer the highest activity toward NRR.

**Figure 7:**
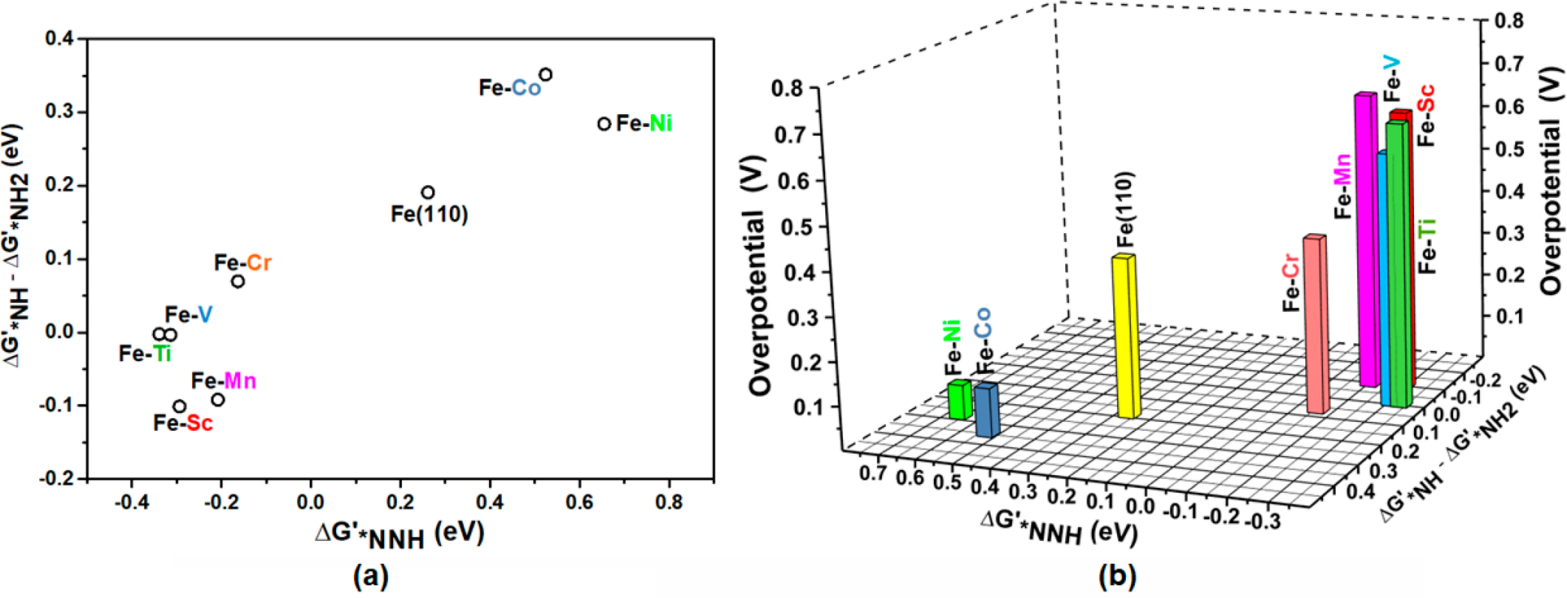
(a) Differences between free energy of adsorption species (Δ*G*′_*NH_ – Δ*G*′_*NH2_) involved in PDS with reference to Δ*G*′_*NNH_ and (b) NRR activity plot for Fe–TM alloys.

To understand the reason behind the variation of the free energy changes over the PDS among the considered alloys, we attempted to scrutinize the structural and electronic effects for some of the important NRR intermediates. In this context, we have determined that the strain present on the surface of the Fe–TM alloys because of the lattice mismatch occurs because two different metal centers Fe and TM are present in these systems. However, the electronegativity difference between them can also play an essential role in fluctuating d-states associated with the surface atoms. The calculated strain effects for Fe–TM alloys and for the pure surface are plotted with reference to strongly binding intermediate *N adsorption energy in Figure S8.^26,65^ The strain analysis shows that half of the Fe–TM (TM = V, Cr, Ni and Co) alloys are under less compressive (more tensile) strain and the other half (TM = Sc, Ti, Mn and Cu) are under more compressive strain with reference to the periodic Fe(110) surface. However, we found that the correlation of *N adsorption energy with the strain effect does not seem to be reasonable. Consequently, the strain effect fails to determine *N adsorption energy as we have found many discrepancies such as lowest compressive strain possessing Fe–Co with weaker adsorption, least adsorption energy over Fe–Cu associated with moderate compression, and strongest binding with Fe–Sc possessing highest compressive strain. Therefore, the first Fe–TM series is less influential in determining the NRR activity for surface alloying. Furthermore, we have also calculated the free energy of adsorption (Δ*G*′) of intermediate species such as *NH, *NH_2_, and *NNH following a favorable associative mechanistic pathway (Text S3).^20^ Adsorption free energy differences of species involved in PDS for alloys and pure surface (Table S8) with respect to adsorption free energy of *NNH species (Δ*G*′_*NNH_) are plotted in Figure 7a.^21^ The overpotential for NRR is plotted as a function of these two quantities as represented in Figure 7b. From their differences, we can understand that *NH formation for alloy surfaces with late TMs (Co and Ni) is less favorable with respect to *NH_2_ formation. Besides, we found (Table 1) that *NH binds weakly on the catalytic surface of late-Fe–TM alloys compared to early-Fe–TM alloys. Therefore, it relatively makes their surface less poisoning in comparison to that of alloying with early-TM and periodic Fe(110) surface as discussed earlier. Moreover, *NH binds strongly on the catalytic surface compared to other *NH_*x*_ and *N_2_H_*x*_ species in all the cases. Furthermore, the observed higher Δ*G*_max_ associated with PDS for early Fe–TM indicates that *NH formation is highly favorable compared to forming *NH_2_. However, opposite trends occur for the surface alloying with late TM in which *NH_2_ formation is favored compared to the formation of *NH. This makes Δ*G*_max_ lower and hence determines the activity. Therefore, enhanced activity was found for late Fe–TM with the plausible formation of *NNH compared to the surface alloying with early Fe–TM and Fe(110) surface, for which the extent of *NNH formation is less facile. Henceforth, inducing late TM over the surface has an enormous impact on improving NRR performances among different iron-based catalytic systems.

**Figure 8:**
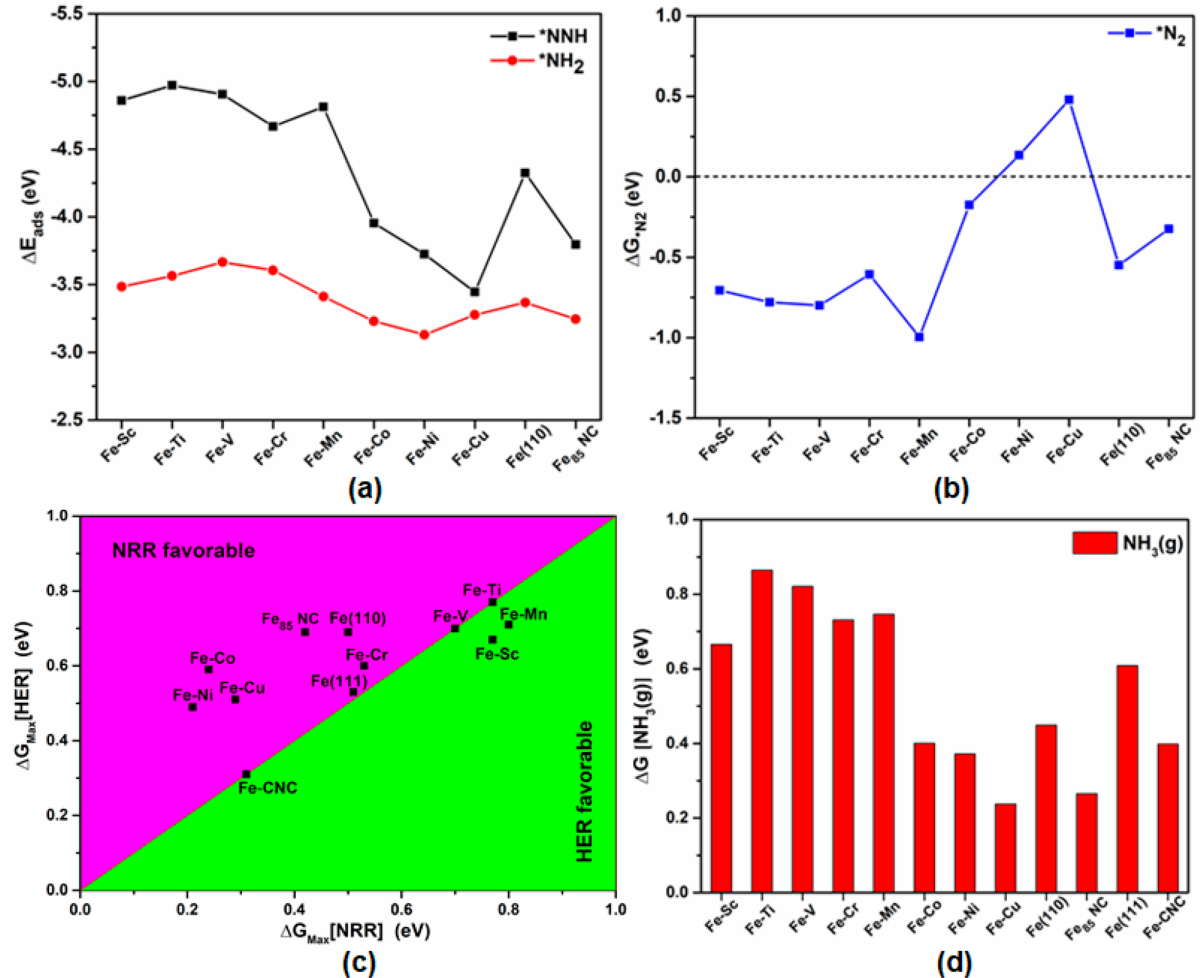
(a) Adsorption energies for *NNH and *NH_2_ species and free energy changes (Δ*G* in eV) associated with (b) activation of the N_2_, (c) NRR (PDS) vs HER (RDS), and (d) desorption energy of ammonia for Fe–TM alloys and other considered systems studied in our earlier report.^26^

Furthermore, the partial density of states (PDOS) has also been analyzed to understand the electronic structure of Fe–TM alloys. We found a comprehensive picture on the discrete binding strength exhibited by their corresponding active site of their surface. We have also calculated the average d-band center value for Fe and TM atoms. The calculated d-band center (ε_d_) values are given in Figure S9. From Figure S9d, it can be seen that the d-band center of Cu atoms for Fe–Cu is more downshifted from the Fermi level compared to other late-TM based alloys. Moreover, the downshifting d-band center value of Co and Ni for Fe–Co and Fe–Ni follows an intermediate trend among the considered alloys, shown in Figure S9. Therefore, we observed an optimum binding strength of the intermediates over Fe–Co and Fe–Ni alloys (Table 1). However, the higher d-state density at the Fermi level (Fe–Co) facilitates a charge transfer of 0.93|e| toward N_2_ in comparison to other considered alloys (Figure S10) such as Fe–Ni (0.86|e|). Henceforth, the activation of *N_2_ over Fe–Co was found to be exergonic (Figure 4a), and sufficient charge transfer (Figure S10) allows it to adsorb with selective stabilization of *NNH, as shown in Figure 8a,b. Among all the candidates, *N_2_ adsorption is less favorable on Fe–Ni and Fe–Cu, as shown in Figure 4b,c and Figure 8b. In addition, the less favorability of *NH formation on the respective site of the Fe–Co alloy prevents the blocking of the sites effectively (Figure 7a) as *NH binds strongly compared to other protonated species (Table 1). Therefore, selective stabilization of *NNH with sufficient *N_2_ activation and destabilization of *NH_2_ for Fe–Co (Figure 8a) reinforces the overall NRR activity following an intermediate trend (Figure 6) between Fe–Ni and Fe–Cu alloys. HER activity for Fe–Co was also found moderate compared to that for other considered systems as discussed earlier. It is evident from Figure 8c that the late TMs highly occupy the NRR favorable region during surface alloying. In contrast, early TMs alloying with the Fe(110) surface, pure Fe(111) surface, and Fe-CNC occupy near the borderline region. So, surface alloying with late TMs can show promising activity toward NRR compared to that with the other considered systems studied in our earlier report. Among them, Fe–Co could be a potential catalyst requiring a Δ*G* of 0.40 eV for NH_3_ formation selectively in the final step, hence removed easily from the surface compared to pure Fe(110), Fe(111) surface, and other considered Fe–TM alloy based catalysts, (Figure 8d). We have also investigated the effect of solvent in the activity trends observed by calculating the reaction free energy changes for the potential determining step (Δ*G*_max_ of PDS) with the inclusion of the solvation effect by using Vaspsol code.^26,46,66−68^ The gas phase versus solvent phase comparison of Δ*G*_max_ for the PDS for Fe–Co and Fe(110) surface are given in Table S9. It can be seen that the solvent effects do not cause significant changes in the energetics of PDS. Also, the observed change in Δ*G*_max_ value along the distal mechanistic pathway is found to be of similar extent for the Fe–Co system and the Fe(110) surfaces. The slight lowering of reaction free energy can be attributed to the stabilization of intermediate species (*NH, *NH_2_) involved in PDS under a solvent environment. Since the solvation inclusion has not caused drastic changes in the energetics, and the higher activity of Fe–Co alloy compared to the Fe(110) surface is still maintained, the results obtained from the gas-phase simulations are expected to be reliable. It is to be noted that the explicit kinetics of the intermediate steps has not been taken into account for the alloy screening in our work. This can be rationalized based on previous studies which have reported congruence between the thermodynamic and kinetic approaches in determining the activity of electrocatalysts. The study by Skúlason et al. on the nitrogen reduction reaction assumed that the activation energy barrier scales with the free energy difference in each of the NRR elementary steps while screening different transition-metal surfaces to construct the activity volcano plot in their study.^20^ Hansen et al. on the oxygen reduction reaction also confirmed that activity volcano constructed for a range of metal surfaces from thermodynamics and kinetics are equivalent.^69^ Recent reports by Asthagiri and co-workers have also shown that the kinetic barrier of the electrochemical CO_2_ reduction reaction follows a similar trend as that of reaction free energy.^70^ Therefore, we have constrained to a thermodynamics-based approach in our study, and we believe our calculated energetics corroborate an electrocatalytic reaction. Though our results provide significant insights toward the composition-dependent NRR activity of the Fe(110) surface, there is plenty of room available for tuning its composition at different levels to obtain further improvement, which will be covered in our future work.

## 4 Conclusion

We have systematically modeled Fe-rich bimetallic Fe–TM alloy-based structural catalysts by incorporating the first series of transition-metal (TM) atoms at different proportions into the periodic Fe(110) surface. The stability analysis from formation energy and average binding energy calculation confirms that all the Fe–TM alloys doping diagonally (model 3D2), except Fe–Cr, Fe–Mn, and Fe–Cu, exhibit reliable stability. It suggests that Fe–TM alloys may withstand the electrochemical condition compared to the pure Fe(110) surface. Moreover, we have carried out the catalytic activity of Fe–TM surface alloy-based structural catalysts toward NRR activity. Our thermodynamic analysis reveals that an associative NRR mechanistic pathway dominates over the dissociative pathway similar to the periodic Fe(110) surface. The identified PDS observed for periodic Fe(110) and Fe–TM (TM = Sc–Ni) is *NH + (H^+^ + e^–^) → *NH_2_, whereas *NH_2_ + (H^+^ + e^–^) → *NH_3_ is the PDS for the Fe–Cu alloy based catalyst. Moreover, the calculated overpotential suggests that late Fe–TM (TM = Co–Cu) can be the active catalysts toward NRR compared to early Fe–TM (TM = Sc–Mn), periodic Fe(110) and Fe(111) surfaces, and also (110) surface of the Fe_85_ nanocluster. This may be due to the favorable *NH_2_ formation on the late-Fe–TM based alloys. Moreover, the low exchange current density associated with HER free energy values suggests that Fe–Co can be the most promising catalyst compared to Fe–Cu and Fe–Ni for NRR. Along with these, the origin of activity differences was explained using electronic structures such as density of states (DOS) and charge transfer analysis for some of the important intermediate species associated with NRR. In addition, Fe–Co is found to be more selective with sufficient *N_2_ activation with plausible *NNH formation toward ammonia production compared to the Fe–Ni and Fe–Cu based structural catalysts. The activity of bimetallic late-Fe–TM alloy catalysts can be developed in the future by substituting other metals which will be the subject of a future work.

## Supporting Information Available

Total energy of all the Fe–TM models, work-function, surface energy average binding energy, formation energy and their corresponding plot, adsorption behavior of all the intermediates, bond length (Fe–M, Fe–Fe) values of the considered models, scaling relationship of *N_2_H_*x*_/*NH_*x*_ (E_*N2Hx/NHx_) species with E_*N_, adsorption energy (E_*N_) values of two *N coadsorption, free energy diagram of dissociative and associative mechanistic pathway, potential determining step, working potential, overpotential values, comparison between associative and dissociative mechanism, elementary steps of hydrogen evolution reaction (HER), overpotential and current density values for HER, plot of *N adsorption energy (E_*N_) for Fe–TM surfaces vs compressive strain, free energy of adsorption (Δ*G*′_*X_), PDOS, d-band center, charge transfer plot and solvation effect. (PDF)
