# Supplementary material for: Computational Screening of First-Row Transition-Metal Based Alloy Catalysts—Ligand Induced N2 Reduction Reaction Selectivity
Source: ACS Phys Chem Au. 2021 Nov 29;2(2):125–35. doi: 10.1021/acsphyschemau.1c00021 (PMC9718324; doi:10.1021/acsphyschemau.1c00021)
Supplement: Supplementary file 1 — pg1c00021_si_001.pdf [file pg1c00021_si_001.pdf]

SUPPORTING INFORMATION

Computational Screening of First-Row Transition-Metal Based Alloy Catalysts–Ligand Induced N_2 Reduction Reaction Selectivity

Arunendu Das,[†] Shyama Charan Mandal,[†] Akhil S. Nair[†] and Biswarup Pathak*,[†]

[†]Department of Chemistry, Indian Institute of Technology Indore, Indore 453552, India

*Email: biswarup@iiti.ac.in

Contents

Table S1. The calculated total energy (E_{total} in eV) values for the following model Fe-TM (TM = Sc-Cu) alloys.

Text-S1. Definition of work-function, surface energy and the following equation for calculation.

Figure S1. The calculated (a) work-function (ϕ) and (b) surface energy values for Fe-TM alloys associated with Model 1C, 2A1, 2D2, 3A1, 3D2 and Fe(110) surface, respectively.

Figure S2. The calculated average binding energy values for Fe-TM alloys associated with Model 1C, 2A1, 2D2, 3A1, 3D2 and Fe(110) surface, respectively.

Figure S3. The calculated (a) formation energy (E_f) and (b) average binding energy (E_b) values for Fe-TM alloys associated with Model 3D and Fe(110) surface, respectively.

Table S2. Adsorption behaviour of the following adsorbed intermediate species for Fe-Co and Fe-Ni surface alloys (Bond length values are given in Å).

Table S3. Bond length (**Fe−TM**, **Fe−Fe**) values for the Fe-TM surface alloys structure and periodic Fe(110) surface.

Figure S4. Scaling behaviour of the adsorption energy of $*N_2H_x$/$*NH_x$ ($E_{*N2Hx/NHx}$) species with reference to E_{*N}.

Table S4. Adsorption energy (E_{*N}) values of co-adsorption of two *N (dissociative adsorption) present at adjacent hollow sites for the Fe-TM surface alloys in different composition.

Figure S5. Free energy profile for dissociative mechanistic pathway following dissociative intermediate species along with ΔG values for surface alloying with early TM and Fe(110).[1]

Figure S6. Free energy profile for associative mechanistic pathway following associative intermediate species along with ΔG values for surface alloying with early TM and Fe(110).[1]

Table S5. ΔG_{max} values with PDS, working potential (U) following dissociative pathway for Fe-TM alloys, Fe(110) surface (studied earlier), respectively.[1]

Table S6. ΔG_{max} values with PDS, working potential (U) and overpotential (η) following associative pathway for Fe-TM alloys, Fe(110) and Fe(111) surfaces, respectively.[1-3,6]

Figure S7. Comparative study of the working potential for NRR following associative and dissociative mechanistic pathway for Fe-TM alloys and Fe(110) surface, respectively.[1]

Text S2. HER elementary steps following Heyrovsky-type mechanistic pathway.

Table S7. The calculated overpotential and current density values for HER on Fe-TM alloys, Fe(110) and Fe(111) surfaces.[1,6]

Figure S8. *N adsorption energy (E_{*N}) of Fe-TM surfaces plotted against compressive strain on the surfaces.

Text S3. Free Energy of Adsorption ($\Delta G'_{*X}$) of the following adsorbed NRR intermediate species.[3]

Table S8. Free energy of adsorption ($\Delta \mathbf{G'_X}$, X = *NNH, *NH and $*NH_2$) and their differences between the species involved in PDS along with overpotential for Fe-TM alloys and periodic Fe(110) surface.[1-3,5]

Figure S9. Partial density of states (PDOS) of surface constituting Fe and TM (a) Mn, (b) Co, (c) Ni and (d) Cu atoms and their d-band center values (ε_d) for Fe-TM alloys.

Figure S10. (a) Charge (|e|) transferred towards N_2 from Fe-TM (TM = Co, Ni, Cu) alloys and Fe(110) surface. (b) Charge variation |e| following associative intermediate species for Fe-Co, Fe-Ni and Fe(110) surface, respectively.

Table S9. Reaction free energy changes for intermediate species involved in PDS on Fe-Co and periodic Fe(110) for gas and solvation (water) medium.[1]

Table S1. The calculated total energy (E_{total} in eV) values for the following model Fe-TM (TM = Sc-Cu) alloys.

**Model**	**Fe-Sc**	**Fe-Ti**	**Fe-V**	**Fe-Cr**	**Fe-Mn**
**1C**	**-288.17**	**-289.79**	**-290.57**	**-290.82**	**-289.42**
**2A1**	**-286.80**	**-290.14**	**-291.63**	**-292.03**	**-290.44**
2A2	-286.80	-290.14	-291.63	-292.03	-----
2D1	-286.89	-290.29	-291.81	-292.23	-289.74
**2D2**	**-287.33**	**-290.44**	**-291.89**	**-292.24**	**-289.75**
2D3	-286.89	-290.29	-291.81	-292.23	-289.74
2D4	-287.33	-290.44	-291.89	-292.24	-289.75
**3A1**	**-283.61**	**-289.64**	**-292.38**	**-292.97**	**-290.20**
3D1	-284.78	-290.45	-292.77	-293.39	-290.10
**3D2**	**-286.58**	**-291.08**	**-293.06**	**-293.43**	**-290.10**

**Model**	**Fe-Co**	**Fe-Ni**	**Fe-Cu**
**1C**	**-288.16**	**-286.74**	**-284.71**
**2A1**	**-287.16**	**-284.29**	**-280.41**
2A2	-287.16	-284.29	-280.41
2D1	-287.21	-284.38	-280.31
**2D2**	**-287.20**	**-284.28**	**-280.25**
2D3	-287.21	-284.38	-280.31
2D4	-287.33	-284.28	-280.25
**3A1**	**-286.10**	**-281.78**	**-276.23**
3D1	-286.26	-282.05	-275.95
**3D2**	**-286.22**	**-281.79**	**-275.68**

Text-S1. Definition of work-function, surface energy and the following equation for calculation.

Work-function (ϕ): The amount of energy needed to eject an electron from metal surface to vacuum, defined as follows,

$$\text{Work-function} = E_{Fermi} - E_{vacuum} \tag{S1}$$

E_{Fermi} is the Fermi energy of the considered slab model and E_{vacuum} is the potential of vacuum region. A system with larger work-function leads to more stable surface.

Surface energy (γ): The amount of energy needed to form a new surface (cleaving an infinite crystal into two parts), defined for considered (Fe-TM) alloys and pure surface as follows,

$$\gamma_{Fe(110)} = E_{surface} - (\beta_{Fe} \times N_{Fe}) \tag{S2a}$$

$$\gamma_{Fe\text{-}TM} = E_{surface} - (\beta_{TM} \times N_{TM} + \beta_{Fe} \times N_{Fe}) \tag{S2b}$$

$E_{surface}$ is the total energy for considered slab model. β_{TM}, β_{Fe} are the bulk energy per atom with their respective crystal structure and N_{Fe}, N_{TM} are the number of Fe and TM atoms for pure Fe(110) and Fe-TM alloys following considered model. A system with large surface energy makes the surface highly reactive.

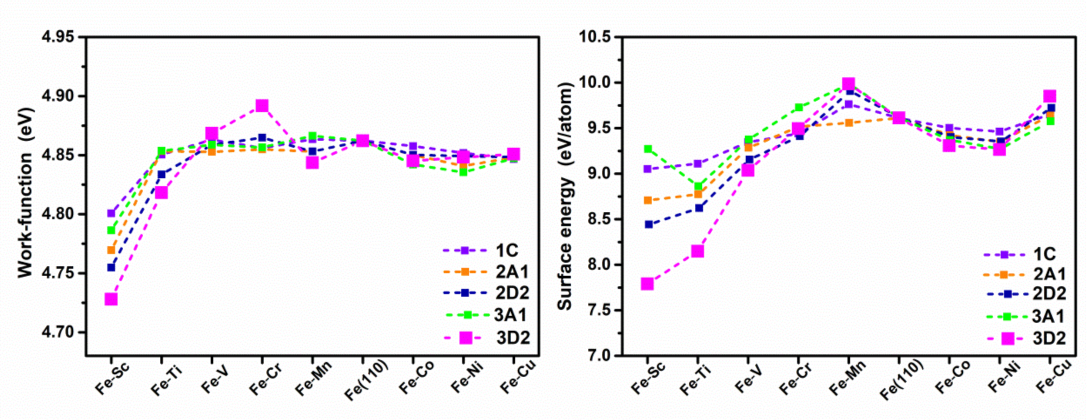

Figure S1. The calculated (a) work-function and (b) surface energy values for Fe-TM alloys associated with Model 1C, 2A1, 2D2, 3A1, 3D2 and Fe(110) surface, respectively.

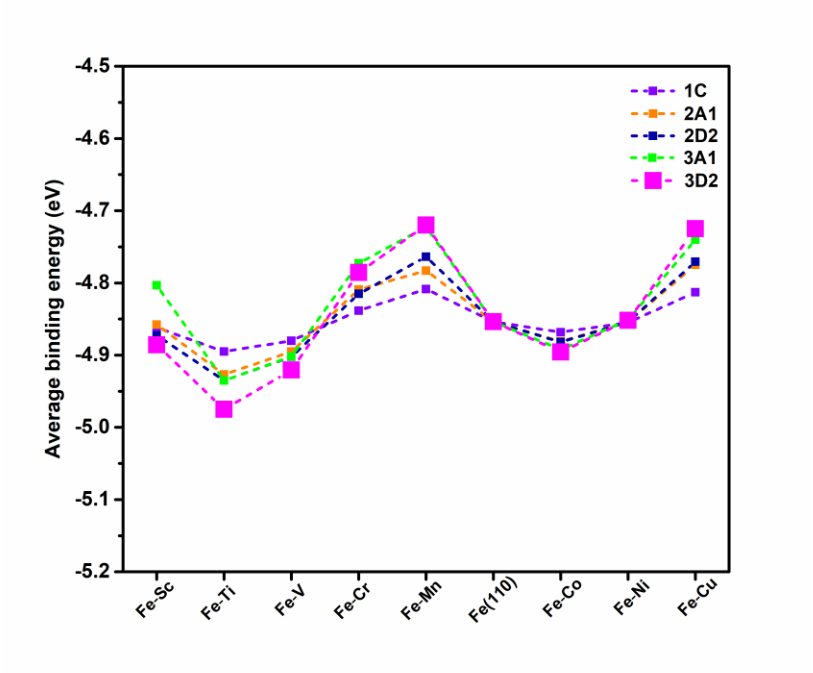

Figure S2. The calculated average binding energy values for Fe-TM alloys associated with Model 1C, 2A1, 2D2, 3A1, 3D2 and Fe(110) surface, respectively.

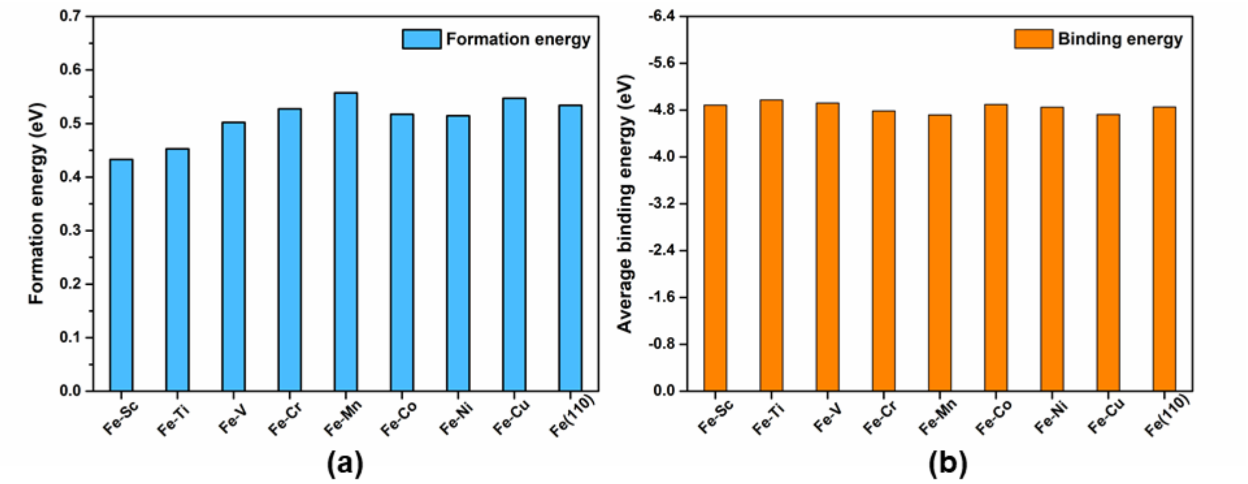

Figure S3. The calculated (a) formation energy ($E_{formation}$) and (b) average binding energy ($E_{binding}$) values for Fe-TM alloys associated with Model 3D and Fe(110) surface, respectively.

Table S2. Adsorption behaviour of the following adsorbed intermediate species for Fe-Co and Fe-Ni surface alloys (Bond length values are given in Å).

Systems with adsorbed species	Fe-Co	Fe-Ni	Elements
$*N_2$	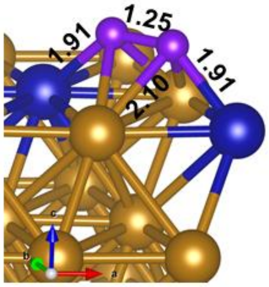	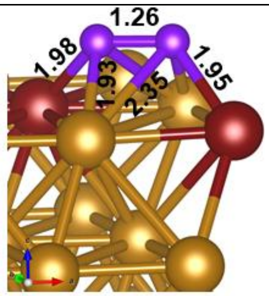	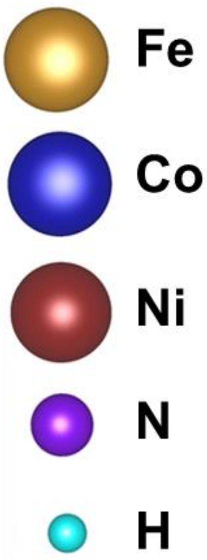
*NNH	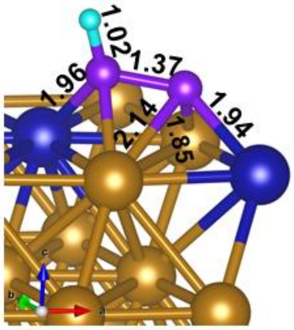	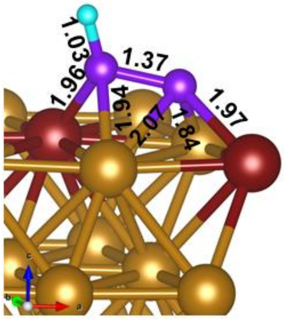	
$*NNH_2$	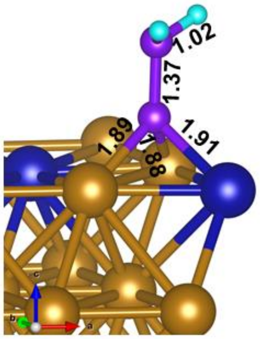	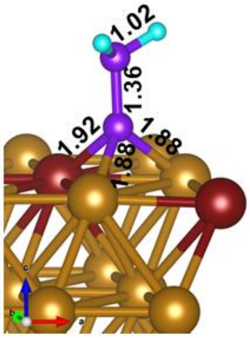	

$*N$	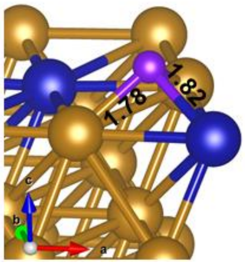 1.78<br>1.82	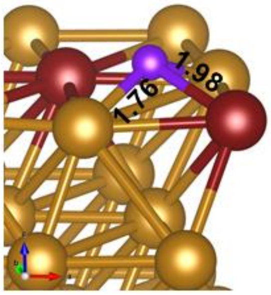 1.76<br>1.98	
$*NH$	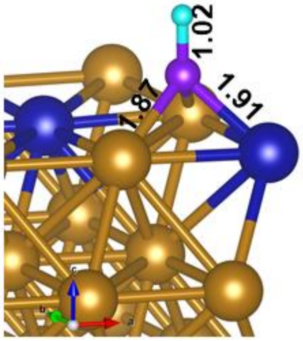 1.02<br>1.87<br>1.91	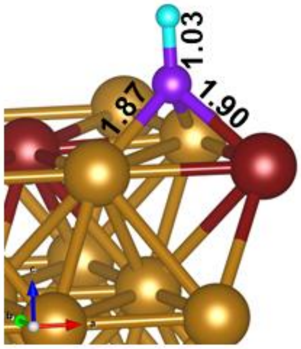 1.03<br>1.87<br>1.90	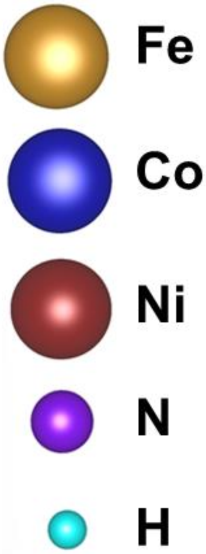 Fe<br>Co<br>Ni<br>N<br>H
$*NH_2$	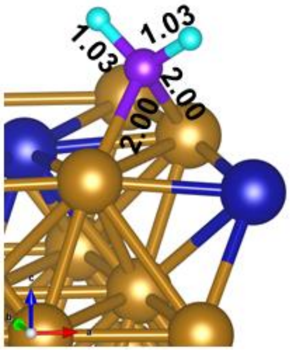 1.03<br>1.03<br>2.00<br>2.00	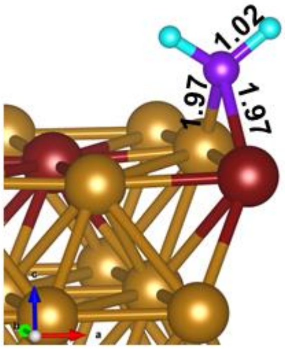 1.02<br>1.97<br>1.97	
$*NH_3$	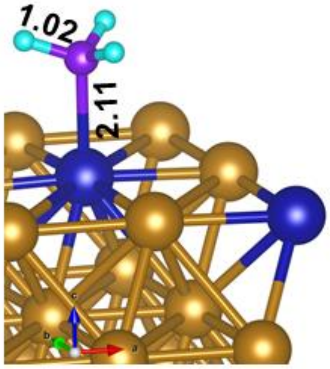 1.02<br>2.11	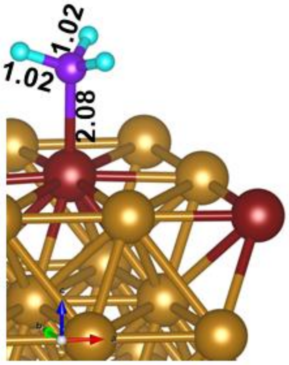 1.02<br>1.02<br>2.08	

*H	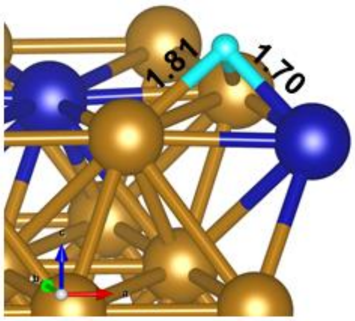	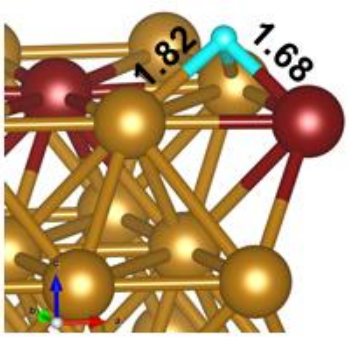	

Table S3. Bond length (**Fe−TM**, **Fe−Fe**) values for the Fe-TM surface alloys structure and periodic Fe(110) surface.[1]

**Systems**	**Bond length (Fe−TM, Fe−Fe) in Å**	
	**SB1 (Fe-TM)**	**SB2 (Fe-Fe)**
**Fe-Sc**	2.57	2.91
**Fe-Ti**	2.45	2.80
**Fe-V**	2.42	2.75
**Fe-Cr**	2.42	2.74
**Fe-Mn**	2.46	2.87
**Fe-Co**	2.41	2.72
**Fe-Ni**	2.42	2.74
**Fe-Cu**	2.44	2.81
**Fe(110)[1]**	2.43	2.77

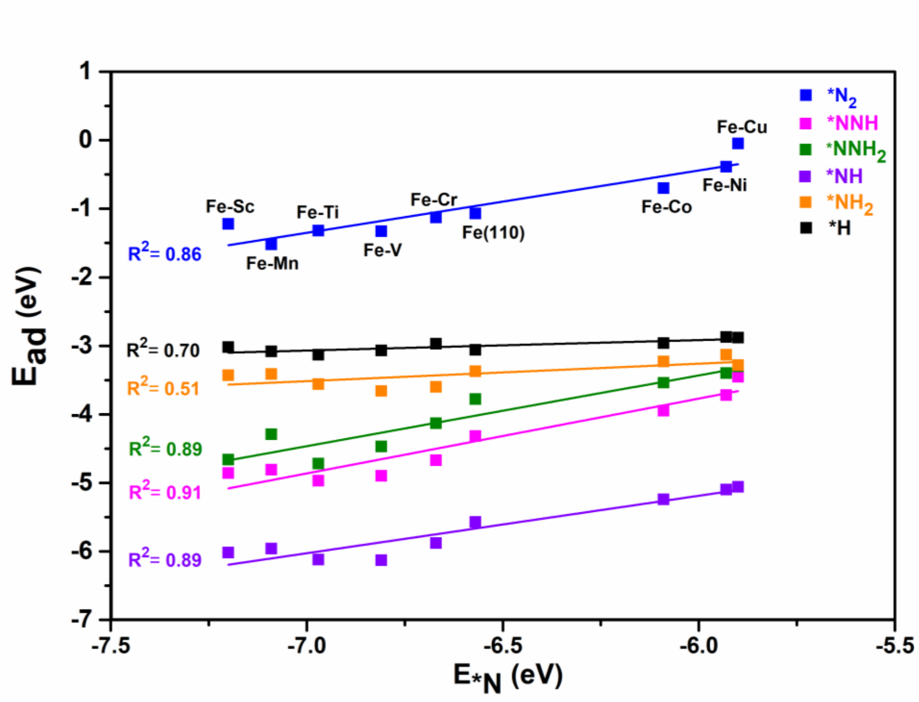

Figure S4. Scaling behaviour of the adsorption energy of $*N_2H_x$/$*NH_x$ ($E_{*N2Hx/NHx}$) species with reference to *N adsorption energy (E_{*N}).

Table S4. Adsorption energy (E_{*N_N}) values of co-adsorption of two *N (dissociative adsorption) present at adjacent hollow sites for the Fe-TM surface alloys in different composition.

**Model**	Adsorption energy (E_{*N_N}) in eV with most stable 3-fold hollow site							
	**Fe-Sc**	**Fe-Ti**	**Fe-V**	**Fe-Cr**	**Fe-Mn**	**Fe-Co**	**Fe-Ni**	**Fe-Cu**
**1C**	-6.92	-6.71	-6.51	-6.45	-6.75	-6.22	-6.17	-6.30
**2A1**	-6.69	-6.55	-6.59	-6.57	-6.53	-6.28	-6.17	-6.13
**2D2**	-7.08	-6.90	-6.69	-6.58	-6.92	-6.12	-6.03	-6.08
**3A1**	-8.06	-6.55	-6.82	-6.86	-7.11	-6.04	-6.00	-5.68
**3D2**	-7.20	-7.12	-6.82	-6.63	-7.07	-6.01	-5.98	-5.95

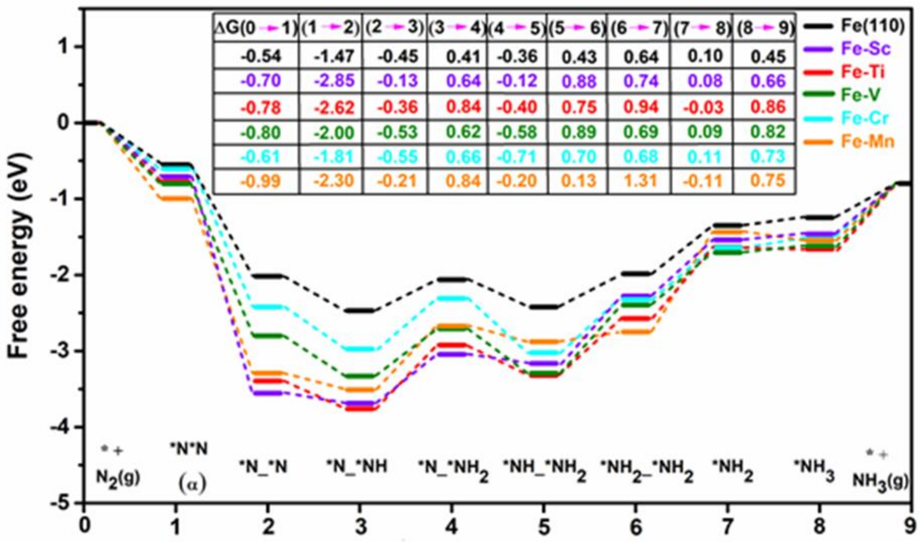

Figure S5. Free energy profile for dissociative mechanistic pathway following dissociative intermediate species along with ΔG values for surface alloying with early TM and Fe(110).[1]

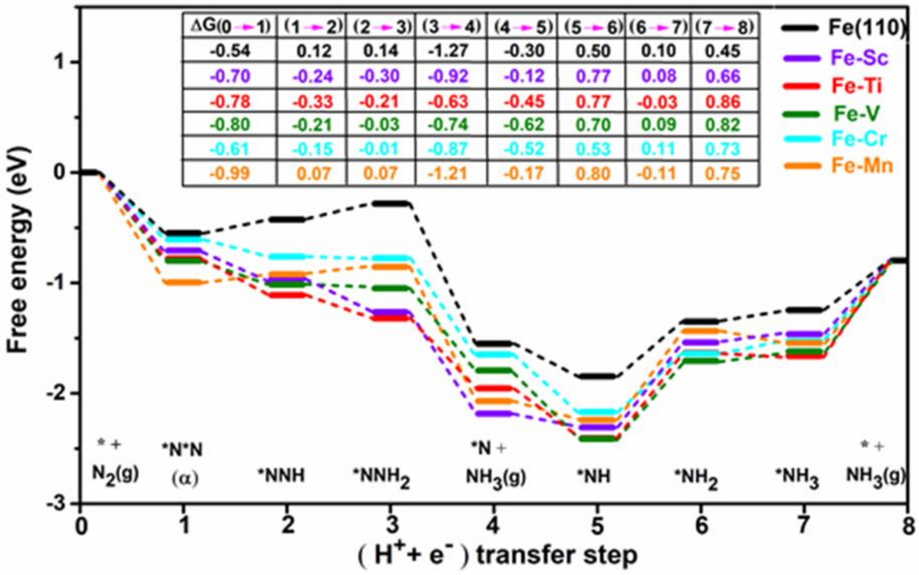

Figure S6. Free energy profile for associative mechanistic pathway following associative intermediate species along with ΔG values for surface alloying with early TM and Fe(110).

Table S5. ΔG_{max} values with PDS, working potential (U) following dissociative pathway for Fe-TM alloys, Fe(110) surface (studied earlier), respectively.[1]

Systems	NRR (Dissociative)		
	PDS	ΔG_{max} (eV)	Working potential U_{work} (V)
Fe-Sc	$*NH\_*NH_2 + (H^+ + e^-) \rightarrow *NH_2\_*NH_2$	0.88	-0.88
Fe-Ti	$*NH_2\_*NH_2 + (H^+ + e^-) \rightarrow *NH_2 + NH_3$ (g)	0.94	-0.94
Fe-V	$*NH\_*NH_2 + (H^+ + e^-) \rightarrow *NH_2\_*NH_2$	0.89	-0.89
Fe-Cr	$*NH\_*NH_2 + (H^+ + e^-) \rightarrow *NH_2\_*NH_2$	0.70	-0.70
Fe-Mn	$*NH_2\_*NH_2 + (H^+ + e^-) \rightarrow *NH_2 + NH_3$ (g)	1.31	-1.31
Fe-Co	$*NH_2\_*NH_2 + (H^+ + e^-) \rightarrow *NH_2 + NH_3$ (g)	0.45	-0.45
Fe-Ni	$*NH_2\_*NH_2 + (H^+ + e^-) \rightarrow *NH_2 + NH_3$ (g)	0.50	-0.50
Fe-Cu	$*NH_2\_*NH_2 + (H^+ + e^-) \rightarrow *NH_2 + NH_3$ (g)	0.55	-0.55
Fe(110)[1]	$*NH_2\_*NH_2 + (H^+ + e^-) \rightarrow *NH_2 + NH_3$ (g)	0.64	-0.64

Table S6. ΔG_{max} values with PDS, working potential (U) and overpotential (η) following associative pathway for Fe-TM alloys, Fe(110) and Fe(111) surfaces, respectively.[1-3,6]

Systems	NRR (Associative)			
	PDS	ΔG_{max} (eV)	Working potential U_{work} (V)	Overpotential (V) $\eta_{NRR} = -0.13 - U_{work}$
**Fe-Sc**	$*NH + (H^+ + e^-) \rightarrow *NH_2$	0.77	-0.77	0.64
**Fe-Ti**	$*NH + (H^+ + e^-) \rightarrow *NH_2$	0.77	-0.77	0.64
**Fe-V**	$*NH + (H^+ + e^-) \rightarrow *NH_2$	0.70	-0.70	0.57
**Fe-Cr**	$*NH + (H^+ + e^-) \rightarrow *NH_2$	0.53	-0.53	0.40
**Fe-Mn**	$*NH + (H^+ + e^-) \rightarrow *NH_2$	0.80	-0.80	0.67
**Fe-Co**	$*NH + (H^+ + e^-) \rightarrow *NH_2$	0.24	-0.24	0.11
**Fe-Ni**	$*NH + (H^+ + e^-) \rightarrow *NH_2$	0.21	-0.21	0.08
**Fe-Cu**	$*NH_2 + (H^+ + e^-) \rightarrow *NH_3$	0.29	-0.29	0.16
**Fe(110)**[1]	$*NH + (H^+ + e^-) \rightarrow *NH_2$	0.50	-0.50	0.37
**Fe(111)**[6]	$*N_2H_3 + (H^+ + e^-) \rightarrow *N_2H_4$	0.51	-0.51	0.38

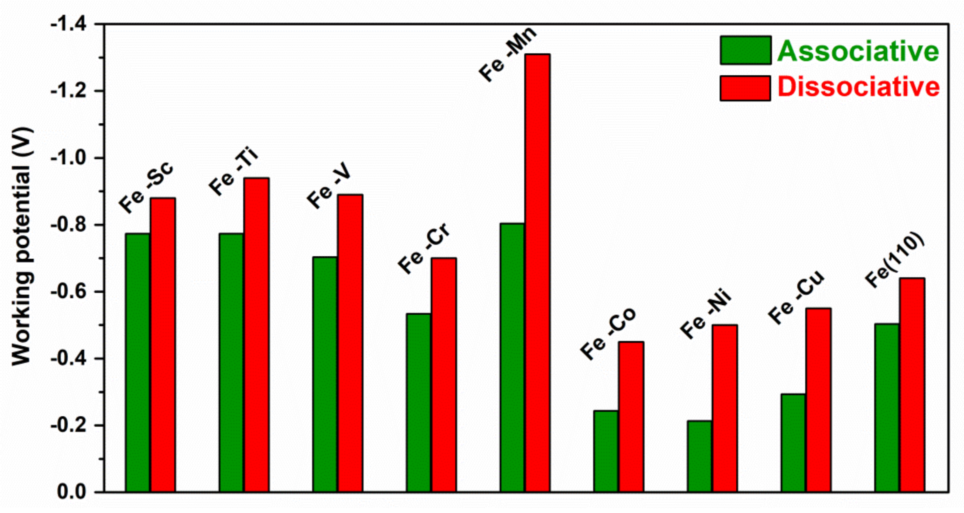

Figure S7. Comparative study of the working potential for NRR following associative and dissociative mechanistic pathway for Fe-TM alloys and Fe(110) surface, respectively.[1]

Text S2. HER elementary steps following Heyrovsky-type mechanistic pathway

$$* + (H^+ + e^-) \rightarrow {}^*H \qquad R1$$

$$^*H \rightarrow (1/2)\, H_2 + * \qquad R2$$

Table S7. The calculated overpotential and current density values for HER on Fe-TM alloys, Fe(110) and Fe(111) surfaces.[1,6]

**Systems**	**HER**				
	**ΔG_{*H} (eV)**	**RDS**	**Overpotential η_{HER} (V)**	**Current density i_0**	**$Log(i_0/Acm^{-2})$**
**Fe-Sc**	-0.667	R2	0.67	6.546×10^{-12}	-11.184
**Fe-Ti**	-0.771	R2	0.77	1.180×10^{-13}	-12.928
**Fe-V**	-0.701	R2	0.70	1.762×10^{-12}	-11.754
**Fe-Cr**	-0.598	R2	0.60	9.397×10^{-11}	-10.027
**Fe-Mn**	-0.713	R2	0.71	1.107×10^{-12}	-11.956
**Fe-Co**	-0.593	R2	0.59	1.140×10^{-10}	-9.943
**Fe-Ni**	-0.494	R2	0.49	5.212×10^{-09}	-8.283
**Fe-Cu**	-0.511	R2	0.51	2.704×10^{-09}	-8.568
**Fe(110)[1]**	-0.688	R2	0.69	2.911×10^{-12}	-11.536
**Fe(111)[6]**	-0.533	R2	0.53	1.135×10^{-09}	-8.945

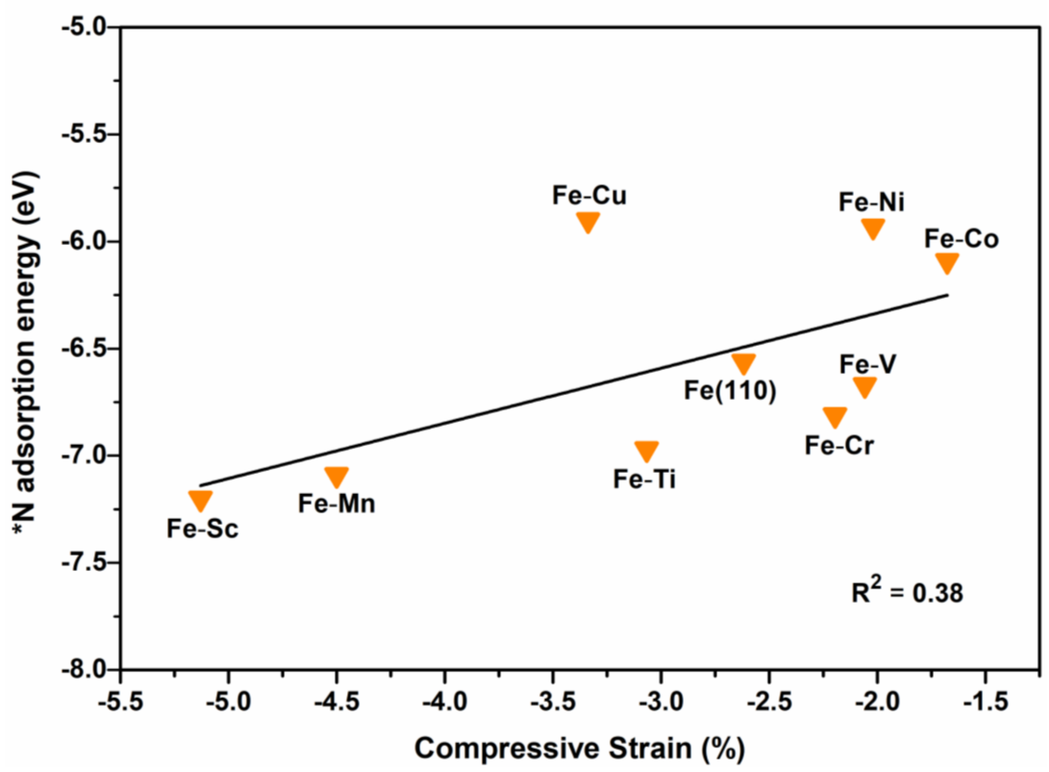

Figure S8. *N adsorption energy of (E_{*N}) Fe-TM surfaces plotted against compressive strain on the surfaces.

The % of strain is calculated by using the following equation,

$$\% \text{ of strain} = (\Delta r/r) * 100$$

Where r is the diameter of the Fe-TM alloys/periodic surface within geometry of optimized structure and Δr is the change in the diameter from their bulk state.[1,4]

Text S3. Free Energy of Adsorption ($\Delta G'_{*X}$) of the following adsorbed NRR intermediate species.[3]

Free Energy of Adsorption ($\Delta G'_{*X}$) of an adsorbed intermediate *NNH, *NH and $*NH_2$ species as follows,

$$N_2(g) + {}^*H \rightarrow {}^*NNH \qquad \Delta G'_{*NNH} = G_{*NNH} - G_{*H} - G_{N2\,(g)} \quad \text{(i)}$$

$$1/2\ N_2(g) + {}^*H \rightarrow {}^*NH \qquad \Delta G'_{*NH} = G_{*NH} - G_{*H} - G_{½\,N2\,(g)} \quad \text{(ii)}$$

$$1/2\ N_2(g) + 2(H^+ + e^-) \rightarrow {}^*NH_2 \qquad \Delta G'_{*NH2} = G_{*NH2} - G_{H2\,(g)} - G_{½\,N2\,(g)} \quad \text{(iii)}$$

Table S8. Free energy of adsorption ($\Delta G'_X$, X = *NNH, *NH and $*NH_2$) and their differences between the species involved in PDS along with overpotential for Fe-TM alloys and periodic Fe(110) surface.[1-3,5]

**Systems**	**NRR**				
	$\Delta G'_{*NNH}$ (eV)	$\Delta G'_{*NH}$ (eV)	$\Delta G'_{*NH2}$ (eV)	$\Delta G'_{*NH} - \Delta G'_{*NH2}$ (eV)	**Overpotential** η_{NRR} **(V)**
**Fe-Sc**	–0.293	–1.243	–1.142	–0.101	0.64
**Fe-Ti**	–0.338	–1.235	–1.233	–0.002	0.64
**Fe-V**	–0.313	–1.311	–1.308	–0.003	0.57
**Fe-Cr**	–0.163	–1.171	–1.241	0.069	0.40
**Fe-Mn**	–0.207	–1.129	–1.037	–0.092	0.67
**Fe-Co**	0.524	–0.530	–0.882	0.351	0.11
**Fe-Ni**	0.654	–0.486	–0.770	0.284	0.08
**Fe(110)**[1]	0.261	–0.760	–0.951	0.191	0.37

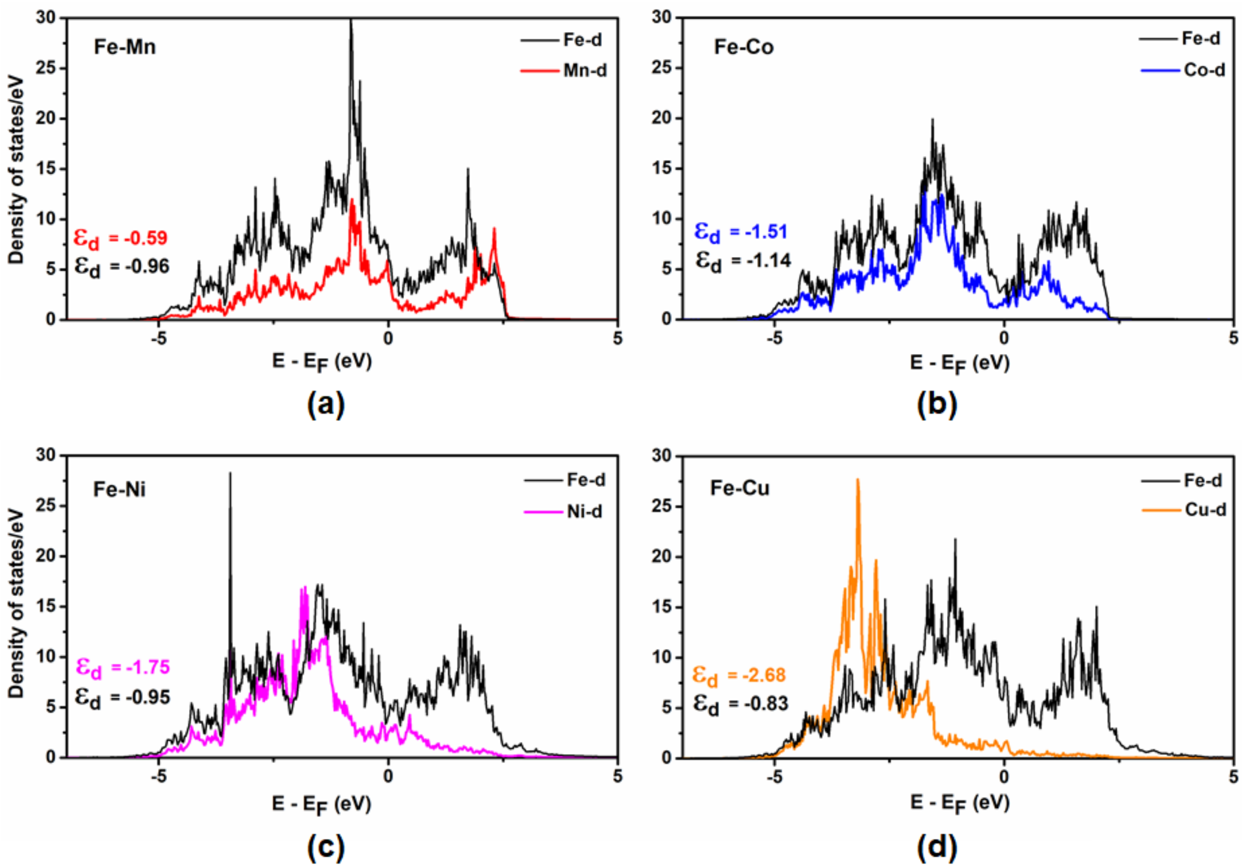

Figure S9. Partial density of states (PDOS) of surface constituting Fe and TM: (a) Mn, (b) Co, (c) Ni and (d) Cu atoms and their d-band center values (ε_d) for Fe-TM alloys.

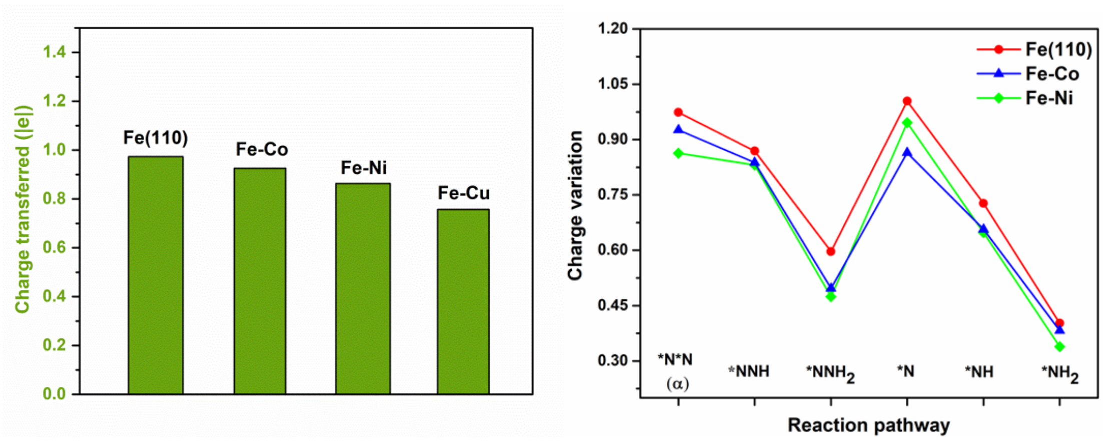

(a) **(b)**

Figure S10. (a) Charge (|e|) transferred towards N_2 from Fe-TM (TM = Co, Ni, Cu) alloys and Fe(110) surface. (b) Charge variation |e| following associative intermediate species for Fe-Co, Fe-Ni and Fe(110) surface, respectively.[1]

Table S9. Reaction free energy changes for intermediate species involved in PDS on Fe-Co and periodic Fe(110) for gas and solvation (water) medium.[1]

**System** (Distal)	**Elementary reaction step involved in PDS**	Gas phase $\Delta\mathbf{G}_{max}$ (eV)	Solvation $\Delta\mathbf{G}_{max}$ (eV)
Fe-Co	$*NH + (H^+ + e^-) \rightarrow *NH_2$	0.24	0.18
Fe(110)[1]	$*NH + (H^+ + e^-) \rightarrow *NH_2$	0.50	0.41

References

1. Das, A.; Nair, A. S.; Mandal, S. C.; Pathak, B. Current Density Calculations of an Octahedral Fe Nanocluster for Selective Electrocatalytic for Nitrogen Reduction. *ACS Appl. Nano Mater*. **2021**, 4, 8, 7758–7770.
2. Zhao, J.; Chen, Z. Single Mo Atom Supported on Defective Boron Nitride Monolayer as an Efficient Electrocatalyst for Nitrogen Fixation: A Computational Study. *J. Am. Chem. Soc.* **2017**, *139*, 12480–12487.
3. Skúlason, E.; Bligaard, T.; Gudmundsdóttir, S.; Studt, F.; Rossmeisl, J.; Abild-Pedersen, F.; Vegge, T.; Jónsson, H.; Nørskov, J. K. A Theoretical Evaluation of Possible Transition Metal Electro-Catalysts for N_2 Reduction. *Phys. Chem. Chem. Phys.* **2012**, *14*, 1235–1245.
4. Kattel, S.; Wang, G. Beneficial Compressive Strain for Oxygen Reduction Reaction on Pt (111) Surface. *J. Chem. Phys.* **2014**, *141*, 124713.
5. Montoya, J. H.; Tsai, C.; Vojvodic, A.; Nørskov, J. K. The Challenge of Electrochemical Ammonia Synthesis: A New Perspective on the Role of Nitrogen Scaling Relations. *ChemSusChem* **2015**, *8*, 2180–2186.
6. Das, A.; Nair, A. S.; Pathak, B. Elucidating Mechanistic Origin of the Catalytic Activity of the Fe(111) Surface and Nanoclusters toward the Electrochemical Nitrogen Reduction Reaction. *J. Phys. Chem. C* **2020**, *124*, 20193–20202.
